# Repertoire-wide phylogenetic models of B cell molecular evolution reveal evolutionary signatures of aging and vaccination

**DOI:** 10.1073/pnas.1906020116

**Published:** 2019-10-21

**Authors:** Kenneth B. Hoehn, Jason A. Vander Heiden, Julian Q. Zhou, Gerton Lunter, Oliver G. Pybus, Steven H. Kleinstein

**Affiliations:** ^a^Department of Pathology, Yale School of Medicine, New Haven, CT 06520;; ^b^Department of Bioinformatics & Computational Biology, Genentech, South San Francisco, CA 94080;; ^c^Interdepartmental Program in Computational Biology and Bioinformatics, Yale University, New Haven, CT 06511;; ^d^Wellcome Centre for Human Genetics, Oxford OX3 7BN, United Kingdom;; ^e^Department of Zoology, University of Oxford, Oxford OX1 3PS, United Kingdom

**Keywords:** B cell repertoire, phylogenetics, BCR, antibody, somatic hypermutation

## Abstract

In order to produce effective antibodies, B cells undergo rapid somatic hypermutation (SHM) and selection for binding affinity to antigen via a process called affinity maturation. The similarities between this process and evolution by natural selection have led many groups to use phylogenetic methods to characterize the development of immunological memory, vaccination, and other processes that depend on affinity maturation. However, these applications are limited by the fact that most phylogenetic models are designed to be applied to individual lineages comprising genetically diverse sequences, while B cell repertoires often consist of hundreds to thousands of separate low-diversity lineages. Further, several features of affinity maturation violate important assumptions in standard phylogenetic models. Here, we introduce a hierarchical phylogenetic framework that integrates information from all lineages in a repertoire to more precisely estimate model parameters while simultaneously incorporating the unique features of SHM. We demonstrate the power of this repertoire-wide approach by characterizing previously undescribed phenomena in affinity maturation. First, we find evidence consistent with age-related changes in SHM hot-spot targeting. Second, we identify a consistent relationship between increased tree length and signs of increased negative selection, apparent in the repertoires of recently vaccinated subjects and those without any known recent infections or vaccinations. This suggests that B cell lineages shift toward negative selection over time as a general feature of affinity maturation. Our study provides a framework for undertaking repertoire-wide phylogenetic testing of SHM hypotheses and provides a means of characterizing dynamics of mutation and selection during affinity maturation.

## Significance

High-affinity antibodies that protect us from infection are produced by B cells through an evolutionary process of mutation and selection during adaptive immune responses. B cell repertoire sequencing combined with phylogenetic methods has provided unprecedented potential to study B cells as an evolving population. However, phylogenetic models operate on individual lineages rather than the thousands of lineages often found in B cell repertoires. Here, we develop an evolutionary framework that incorporates B cell-specific features and combines information across lineages to characterize mutation and selection dynamics of entire repertoires. We use this technique to demonstrate evidence of age-associated changes in somatic hypermutation targeting and uncover a general trend within our datasets toward negative selection over the course of affinity maturation.

B cell receptors (BCRs) are membrane-bound immunoglobulins (Ig) expressed on the surfaces of B cells that bind to antigen and may be released as antibodies to fight infection. BCRs are generated through the shuffling of Ig gene segments by V(D)J recombination and, if the cell expressing them is activated, by a second process of BCR modification called affinity maturation (1). Affinity maturation consists of repeated rounds of somatic hypermutation (SHM) of the BCR, cell proliferation, and selection for antigen binding affinity (1). These processes give rise to clonal lineages of B cells that each descend from a progenitor cell, from which they differ predominately by point mutations. The BCR sequence, and the nature of the mutations introduced during affinity maturation, can be investigated in detail using high-throughput next-generation BCR sequencing (2–4).

The fact that affinity maturation is a form of evolution by natural selection suggests that methods from molecular evolutionary biology, particularly phylogenetics, could have broad utility in studying affinity maturation. This has stimulated the development of methods of evolutionary sequence analysis designed specifically for BCR sequences, particularly phylogenetic approaches (5–8). These methods have shown promise in elucidating information about the adaptive immune response in humans, such as the sequence of mutations that occur during antibody coevolution with HIV (9) and the migration of B cells in multiple sclerosis (10).

There are a number of challenges in adapting phylogenetic techniques to B cell clonal lineage analysis. Phylogenetic techniques are typically used to analyze individual, genetically diverse B cell lineages (9, 11). However, B cell repertoires are typically profiled by next-generation sequencing and consist of many—potentially thousands of—expanded clonal lineages, each of which may contain only a few unique sequences (12, 13). In light of this, many analyses have used nonphylogenetic summary statistics to characterize BCR repertoires, such as the distribution of sequences per clone (14–16). For example, 2 recent studies have used frequency-based statistics (17) and a generalized McDonald–Kreitman test (18, 19) for characterizing B cell selection. Other nonphylogenetic approaches have been developed to study SHM biases (20) and signatures of clonal selection (21) by representing B cell clonal lineages using a single representative sequence. By explicitly modeling shared ancestry among sequences within the same clone, phylogenetic approaches offer a potentially more powerful means of understanding SHM and affinity maturation by using the full set of substitutions inferred to have occurred in a repertoire. However, standard phylogenetic approaches are limited to single lineages and give imprecise parameter estimates, except when applied to unusually large or highly diverse B cell lineages (7). Even when analyzing individual clonal lineages, the biology of affinity maturation violates fundamental assumptions in most phylogenetic substitution models, such as independent change at each nucleotide site and time reversibility of substitution rates (22).

We propose here that it is possible to combine some of the benefits of phylogenetic and summary statistic approaches of B cell repertoire analysis by using hierarchical phylogenetic models. These approaches contain multiple levels of parameters, some of which are shared among lineages, while others are estimated for each lineage individually (23). For example, Rodrigo et al. (24) applied one such hierarchical phylogenetic approach to a set of HIV sequences from infected patients in order to jointly estimate both the virus substitution rate and the proportion of individuals that did not respond to antiretroviral therapy. However, previous applications of hierarchical phylogenetic models to virus genomes do not address the abovementioned model assumptions that are violated by the biology of B cell affinity maturation.

A hierarchical approach that is specifically tailored to B cell sequence evolution has the potential to dramatically improve accuracy of parameter estimation. Toward this end, we propose a “repertoire-wide” phylogenetic framework, a hierarchical approach in which all parameters are constrained to be identical among lineages within a repertoire. By assuming that B cell lineages within a particular repertoire experience broadly similar patterns of substitution (e.g., hot- and cold-spot sequence motifs that experience altered mutation rates under SHM), a repertoire-wide approach is able to share information across B cell lineages and thereby take advantage of the genetic diversity of the entire repertoire, despite the fact that each individual lineage within the BCR repertoire data may exhibit low diversity. This repertoire-wide phylogenetic framework is capable of characterizing entire B cell repertoires by jointly estimating parameters and lineage tree topologies for all lineages within a repertoire. We first introduce a phylogenetic substitution model that accounts for both context-sensitive mutation and changing codon frequencies during affinity maturation and validate our repertoire-wide approach through simulation. We then apply this framework to characterize the effects of aging on B cell repertoire development and B cell responses to influenza vaccination. We demonstrate that repertoire-wide approaches can quantify variation in SHM features both across individuals and within the same individual through time. Our results reveal previously uncharacterized immunological phenomena underlying aging and vaccination. We discover 1) evidence of changes in SHM hot-/cold-spot mutation biases associated with age, 2) evidence of negative selection acting on complementarity-determining regions (CDRs) associated with the human immune response to influenza vaccination, and 3) a consistent relationship between increased lineage tree length and signatures of negative selection across our datasets.

## Methods

### A Nonstationary, Nonreversible Phylogenetic Substitution Model for B Cell Evolution.

The process of nucleotide change along a given phylogenetic tree is modeled as a Markov process, such that the rate of transitioning into any state at each instant in time is dependent only on the current state of the model (11). Here, we characterize codon change in Ig sequences using the HLP19 substitution model (*SI Appendix*, section S1), a 61- × 61-element matrix (**Q** matrix) that describes the instantaneous rates of change between all nonstop codons. These instantaneous rates are parameterized by the nonsynonymous/synonymous mutation rate ratio (*ω*), transition/transversion mutation rate ratio (*κ*), a vector of 61 nonstop codon frequencies (**π**), and a vector of modified substitution rates **h** = (*h*^*WRC*^, *h*^*GYW*^ …, *h*^*(a)*^) where each value *a* is an SHM hot- or cold-spot motif, such as WRC (ref. 25; W = A/T, R = A/G; only the underlined base experiences increased substitution).

Most phylogenetic substitution models make a salient approximation that nucleotide or codon frequencies are constant over time at a stationary distribution (26). However, the codon composition of B cell sequences begins substantially far away from equilibrium and changes over time (27), making this assumption inappropriate. A previous model of affinity maturation, HLP17 (7), attempted to address this problem by using maximum likelihood (ML) to estimate codon frequencies. While this approach may be better than empirical estimates of codon frequencies, at least in some instances, it more than doubles the number of model parameters. In contrast, the HLP19 model introduced here (*SI Appendix*, section S1) uses the predicted codon frequencies at the midpoint of phylogeny in question. Overall, HLP19 has less than half the number of free parameters as HLP17 and exhibits improved branch length estimates, generally better estimates of certain substitution model parameters such as *ω* (*SI Appendix*, section S3), and significantly improved run time and is structurally more similar to other nonreversible substitution models (28, 29).

### Repertoire-Wide Phylogenetic Models.

Under standard ML phylogenetics (11), a single multiple sequence alignment **X** is specified, and the goal is to find the tree topology and branch lengths **T**, and the set of substitution parameters, that maximize the likelihood of **X**. For B cell lineage phylogenies, the sequence alignment is supplemented with a predicted germline sequence **G** that acts as an outgroup and adds direction to the tree. In this study we extend this approach by calculating the likelihood of the entire B cell repertoire, which we define as the product of the tree likelihoods for each of *n* lineages, using each lineage *i*’s tree topology (**T**_*i*_), substitution parameters (*ω*_*i*_, *κ*_*i*_, **h**_*i*_), sequence data (**X**_*i*_), and predicted germline sequence (**G**_*i*_) (Eq. **1**). This approach therefore assumes that mutations in each lineage are independent from each other:

$$\begin{array}{c} L_{repertoire}=\prod_{i=1}^{n}L\left(T_{i},\omega_{i},\kappa_{i},h_{i}|X_{i},G_{i}\right) \end{array}.$$ [1]

The goal of our phylogenetic repertoire analysis is to find the tree topologies, branch lengths, and substitution parameters that maximize Eq. **1**, whereas the goal of typical ML phylogenetic analysis is to maximize individually each phylogenetic likelihood on the right-hand side. In a repertoire-wide model, parameters are constrained to be identical across lineages, allowing them to be estimated at the repertoire level. For instance, we may estimate a repertoire-wide transition/transversion rate ratio by constraining *κ*_*1*_ = *κ*_*2*_ = … *κ*_*n*_. Constraining parameters in this way will lower the overall likelihood of the repertoire compared to optimizing parameters for each lineage individually (because there will be fewer degrees of freedom) and will mask any true variation among lineages but will decrease the number of parameters and thereby reduce parameter estimation variance. For the analyses presented here, we constrain all substitution parameters to be identical across lineages within a repertoire.

### B Cell Repertoire Datasets.

We use repertoire-wide phylogenetic models to characterize B cell repertoires in 2 previously published datasets obtained from peripheral blood samples. The first dataset (Age) consists of samples taken from 27 healthy individuals without any known recent infections or vaccinations in 2 consecutive years (30). Subjects varied in age from 20 to 81 years old and both male and female subjects were included. Our second dataset (Vaccine) consists of samples from 3 male donors aged 30 (subject hu420139), 34 (420IV), and 55 (PGP1) years old at 10 time points: −8 d, −2 d, −1 h, +1 h, +1 d, +3 d, +7 d, +14 d, and +28 d relative to seasonal influenza vaccination (31). Each of these sequence datasets was produced from total messenger RNA from unsorted peripheral blood mononuclear cells. Quality control and data processing for both of these datasets is detailed in *SI Appendix*, section S1. Samples from each time point in the Vaccine dataset had between 141 and 15,763 (mean 6,272.7) unique sequences in nonsingleton clones (i.e., clones containing >1 unique sequence). Because of this large variation and the computational complexity of our methods, the repertoires in the Vaccine dataset were subsampled to a depth of 3,000 sequences in nonsingleton clones (*SI Appendix*, section S1). Sequence depth in the Age dataset was more even, with between 370 and 2,065 (mean 1,126) unique sequences in nonsingleton clones, so the repertoires in the Age dataset were not subsampled.

### Phylogenetic Model Parameter and Topology Estimation.

We used a single-linkage hierarchical clustering approach, detailed in *SI Appendix*, section S1, to assign sequences into clonal lineages, each of which was assumed to descend from a single naïve B cell ancestor. Because we were not able to reliably predict the junction regions of germline sequences (32), we removed the CDR3 from all sequences analyzed. We then used the repertoire-wide phylogenetic model described above to quantify effects of BCR mutation and selection in the Age and Vaccine datasets.

Phylogenetic model parameters are an important source of information about evolutionary dynamics. For example, the amino acid replacement vs. silent mutation rate ratio (*ω*) can be used to distinguish positive and negative selection (33), while the relative rate of transitions to transversions (*κ*) can be informative about mutation biases. We first estimated ML tree topologies and branch lengths for each B cell lineage using the GY94 (33, 34) substitution model, in which single, shared *ω* and *κ* parameters were estimated for each repertoire, and codon frequencies were set to their empirical frequencies across all sequences within each repertoire. For computational efficiency, we used these estimated topologies to estimate branch lengths and substitution parameters of the HLP19 model at the repertoire level; namely, we estimated *κ*, *ω*_*FWR*_, and *ω*_*CDR*_ [separate *ω* values for CDRs and framework regions (FWRs)] and **h** values (altered relative mutation rate) for WRC, GYW, WA, TW, SYC, and GRS hot- and cold-spot motifs (see *SI Appendix*, section S1 for details on these parameters).

Hypotheses concerning substitution model parameter estimates can be tested in a phylogenetic framework using a likelihood ratio test (35). For models that differ only by one free parameter, a *P* value of 0.05 corresponds to a log-likelihood difference of 1.92 between the alternative (ML estimated) and null (fixed value) model (35). The log-likelihood ratio test allows estimation of 95% CIs for parameter estimates using profile likelihood curves. Each point on a profile likelihood curve is created by calculating the ML obtained when the parameter of interest is fixed to a particular value and all other parameters are optimized. We used a straightforward binary search approach to estimate the 95% CI either side of the ML estimate.

### Dataset Simulation.

As a means of validation, simulations (detailed in *SI Appendix*, section S2) were performed to test 1) the performance of the HLP19 model relative to the previous HLP17 and GY94 models (*SI Appendix*, section S3) and 2) the effects of estimating parameters using a repertoire-wide phylogenetic model compared to inference from individual lineage trees (*SI Appendix*, section S4). To verify that the trends we observe in the Age and Vaccine datasets are not simply the result of biases in our parameter estimation procedure, we performed simulations using prespecified substitution parameters (*κ* = 2, *ω*_*FWR*_ = 0.5, *ω*_*CDR*_ = 0.7, *h*^*WRC*^ = 4, *h*^*GYW*^ = 6, *h*^*WA*^ = 4, *h*^*TW*^ = 2, *h*^*SYC*^ = −0.6, and *h*^*GR*S^ = −0.6) and the same tree topologies and branch lengths as the empirical trees from the Age and Vaccine datasets. We then repeated the analyses performed in each section on these simulated datasets (*SI Appendix*, section S6). We further compared model performance under simulations employing the S5F empirical model of SHM motif mutability (20). Again, empirical tree topologies and branch lengths were used during simulation (*SI Appendix*, section S7).

## Results

### Repertoire-Wide Phylogenetic Models Improve Parameter Estimation.

Phylogenetic substitution model parameters can be an important source of information about the evolutionary dynamics of lineages; for instance, the amino acid replacement vs. silent mutation rate ratio (*ω*) is used to characterize natural selection operating on genetic sequences (33). The HLP19 model parameters are informative about the process of B cell affinity maturation. The model includes separate *ω* parameters for the FWRs and CDRs (*ω*_*FWR*_ and *ω*_*CDR*_), the transition/transversion rate ratio (*κ*), and a set of altered substitution rates at SHM hot-/cold-spot motifs (*h*^*WRC*^, *h*^*GYW*^, *h*^*WA*^, *h*^*TW*^, *h*^*SYC*^, and *h*^*GR*S^; nucleotides represented using the International Union of Pure and Applied Chemistry coding scheme, only underlined bases experience altered rates).

The small size of most B cell lineages poses a problem for accurate estimation of phylogenetic model parameters for individual B cell lineages. Namely, the size distributions of clonal lineages within B cell repertoires, particularly those derived from blood samples, typically follow a power-law distribution and are dominated by many lineages that each carry only a few unique sequences (12, 13). We confirmed this pattern using blood sample-derived BCR repertoires from 27 healthy subjects (Age dataset; ref. 30). Across these subjects 88 to 96% (mean: 92.3%) of lineages comprised a single unique sequence, and between 98 and 99.8% (mean: 99.3%) of lineages contained <5 unique sequences.

**Fig. 1.**
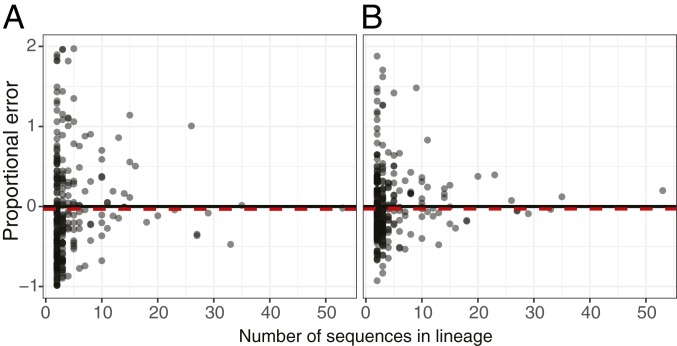
Proportional error of repertoire-wide and individual lineage estimates. (*A*) Proportional error in estimates of the *ω*_*CDR*_ parameter under the HLP19 model. (*B*) Proportional error in estimates of the *ω*_*FWR*_ parameter under the HLP19 model. In *A* and *B* the black dots show the values estimated from each individual lineage B cell lineage and the red dotted lines show the estimate obtained from all lineages combined using a repertoire-wide model. Data were generated from a simulated repertoire using tree topologies from subject 97 in the Age dataset and identical parameters among lineages (see *SI Appendix*, section S4 for full details and results). Note that 14% and 3% of lineages for *A* and *B*, respectively (all with ≤18 sequences), had proportional error higher than the range displayed in these plots. See *SI Appendix*, Fig. S4*D* for the full range.

Mutation and selection in B cell lineages can be analyzed at multiple levels; we may be interested in the dynamics of specific lineages or in repertoires as a whole. Individual lineages may be characterized using the parameter estimates of a substitution model; however, these estimates will be highly inaccurate for small lineages (Fig. 1), which typically make up the majority of lineages in a repertoire (12, 13). Whole B cell repertoires may be characterized by estimating model parameters for each lineage individually and then averaging these values across lineages (hereafter termed the mean individual estimate), although these estimates will still be affected by the inaccuracy of small lineages. Alternatively, we propose to link all lineages within a repertoire to estimate a single set of repertoire-wide parameter values (*Methods*). This approach has the potential to reduce the error and variance of parameter estimates used to characterize B cell repertoires.

We used a model of SHM and empirically derived tree topologies to simulate realistic repertoire datasets and thereby test the performance of our approach (*SI Appendix*, section S4). Simulated datasets consisted of 289 lineages in total (≥2 sequences) of which 34 lineages had ≥10 sequences and 4 lineages had ≥30 sequences. We first simulated datasets with identical parameters among lineages and then reestimated HLP19 model parameters at the repertoire and individual-lineage level. Repertoire-wide estimates had lower variance compared to mean individual estimates in all comparisons performed. Averaging across all parameters, repertoire-wide estimates showed lower bias (repertoire-wide = −0.04, best mean individual = −0.05), variance (repertoire-wide = 0.01, best mean individual = 0.16), and mean squared error (MSE; repertoire-wide = 0.11, best mean individual = 0.29) than mean individual estimates (*SI Appendix*, Table S4B). Further, repertoire-wide estimates had lower MSE than mean individual estimates in all instances except one (mean *h*^*GYW*^ estimates from lineages with ≥10 sequences; *SI Appendix*, Table S4B). In contrast, repertoire-wide estimates had lower bias for only 4 of 9 parameters when compared to mean individual estimates obtained from larger lineages (≥10 or ≥30 sequences). Thus, under these simulation conditions repertoire-wide estimates are superior to mean individual estimates. Repertoire-wide estimates were not always less biased than mean individual estimates from large lineages, but they were less variable and had lower overall error rates.

We next relaxed the assumption that all lineages have the same parameter values by performing simulations in which *ω*_*CDR*_ and *ω*_*FWR*_ varied among lineages (*SI Appendix*, section S4). As before, repertoire-wide estimates of *ω*_*CDR*_ and *ω*_*FWR*_ had substantially lower bias, variance, and MSE compared to mean individual estimates obtained by averaging across all lineages. Repertoire-wide estimates also had lower variance and MSE than mean individual estimates obtained from larger lineages (i.e., ≥10 or ≥30 sequences), but not always lower bias (*SI Appendix*, Table S4C). We also tested how well repertoire-wide estimates characterized lineage-specific values of *ω*_*CDR*_ and *ω*_*FWR*_ (while constraining all lineages to have the same parameter values reduces variance we hypothesized it may introduce a bias at the lineage level). Surprisingly, repertoire-wide estimates of lineage-specific *ω*_*CDR*_ and *ω*_*FWR*_ were less biased than mean individual estimates when all lineages within the repertoire were considered. However, estimates of lineage-specific parameters obtained from larger lineages (≥10 and ≥30 sequences) were less biased than repertoire-wide estimates (*SI Appendix*, Table S4C). Overall, we find that a repertoire-wide phylogenetic approach has substantial benefits even when the underlying parameters vary among lineages.

To test the strengths and weaknesses of different phylogenetic models in our repertoire-wide framework, we compared the performance of 3 codon substitution models: GY94 (33, 34), which does not include SHM hot- or cold-spot motifs, HLP17 (7), which is a modification of the GY94 model that incorporates hot- and cold-spot biases, and HLP19, which is introduced herein (*SI Appendix*, section S1) that differently incorporates the dynamics of codon frequencies during affinity maturation and is more formally similar to previous nonreversible models (29). In HLP19, the relative probability of a substitution depends only on whether the substitution is a replacement or silent mutation, a transition or transversion, and its probability of occurring in an SHM motif (*SI Appendix*, section S1), whereas in HLP17 substitution from codon *a* to codon *b* additionally depends on the frequency of codon *b*. Simulation analyses performed using multiple tree topologies and parameter values (*SI Appendix*, section S3) revealed that parameter estimates under HLP19 had a lower mean absolute bias across all parameters (0.03) than HLP17 (0.08) and GY94 (0.16; *SI Appendix*, section S3). HLP19 and GY94 models had similar absolute bias in branch lengths (<0.002), which was lower than that of HLP17 (0.11; *SI Appendix*, section S3). HLP17 performed worse than GY94 in branch-length estimation, which is surprising given that Hoehn et al. (7) showed that the HLP17 model improved branch-length estimates compared to GY94. However, we have since determined that the simulations performed in ref. 7 were unintentionally but unfairly biased toward the HLP17 model (see detailed explanation in *SI Appendix*, section S2). The simulations performed here do not have this issue and show that HLP19 largely addresses the weaknesses of on HLP17 in branch-length estimation (*SI Appendix*, section S3). Perhaps most importantly, for parameters relating to selection (*ω*_*FWR*_ and *ω*_*CDR*_) HLP19 showed significantly lower mean absolute bias (0.02) compared to HLP17 (0.1) and GY94 (0.29; *SI Appendix*, section S3). Mean bias of *ω*_*CDR*_ estimates were especially high under the GY94 model (range: 0.38 to 0.59) and increased in simulations with higher hot-spot mutation rates and longer branch lengths (*SI Appendix*, section S3). This echoes previous findings; models that fail to account for altered mutation rates of SHM motifs (e.g., GY94) can significantly bias estimates of *ω* (dN/dS) in BCR lineages toward detecting positive selection in the CDRs (36, 37). Simulations under an empirical model of SHM context sensitivity (20) and empirically estimated tree topologies confirm that *ω*_*CDR*_ and *ω*_*FWR*_ estimates from HLP19 remain less biased than estimates under HLP17 and GY94 under alternative substitution regimes (*SI Appendix*, section S7). Overall, we found the HLP19 model shows superior performance compared to the GY94 and HLP17 models, particularly when estimating *ω*_*CDR*_ and branch lengths, respectively.

To further compare the appropriateness of the GY94, HLP17, and HLP19 models when applied to BCR repertoire data, we estimated how well each model fit our empirical datasets using the Akaike information criterion (AIC; ref. 38). The AIC uses the maximum log-likelihood estimated using a model, penalized by the number of freely estimated parameters. Smaller AIC values are generally interpreted as better model fit. To make AIC values comparable among the 3 models, we altered the HLP17 and HLP19 models slightly by multiplying the partial likelihood of each possible codon at the root by the frequency of that codon (*π*), as is typically done for reversible models (11). In all 27 subjects AIC was highest under GY94 and lowest under HLP19, indicating that the HLP19 model had a significantly better fit to all subjects compared to the GY94 and HLP17 models (*SI Appendix*, section S5).

### Variation of Model Parameters within and among Subjects.

**Fig. 2.**
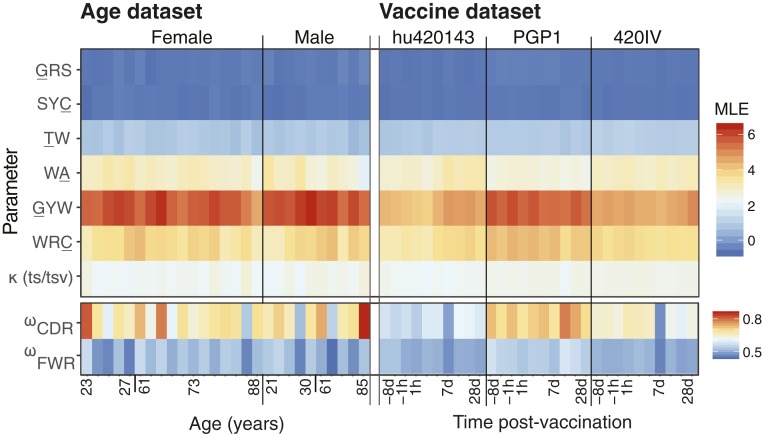
Variation of parameter estimates by subject and time in the Age and Vaccine datasets. (*Left*) HLP19 parameter estimates from each subject in the Age dataset, ordered by sex and age. (*Right*) HLP19 parameter estimates for the Vaccine dataset, ordered by subject and sample time relative to influenza vaccination. The upper box in both panels shows the model parameters that relate to SHM (motif targeting, transition/transversion ratio). The lower box shows estimates of *ω*_*CDR*_ and *ω*_*FWR*_, which relate to selection. The 95% CIs for these estimates are shown in *SI Appendix*, Fig. S10. Note that values in the lower box are scaled differently from those in the upper box (see keys on the right-hand side).

We tested whether repertoire-wide parameter estimates can reproduce known features of SHM targeting, such as hot-/cold-spot targeting (20) by estimating HLP19 model parameters from BCR repertoire data that were obtained from 27 healthy individuals of varying age and sex (Age dataset; ref. 30). While the values of parameter estimates varied, all subjects exhibited the same overall pattern in model parameters that relate to SHM targeting (Fig. 2). In all subjects, GYW motifs exhibited the largest substitution rate increases of the all motifs considered (*h*^*GYW*^ values were 4 to 6), followed by the WRC (*h*^*WRC*^ ∼3), WA (*h*^*WA*^ ∼3), and TW (*h*^*TW*^ ∼1) motifs. Symmetrical SYC and GRS motifs were estimated to be mutational cold spots (*h*^*SYC*^ and *h*^*GRS*^ ∼ −0.6). We compared these parameter estimates to mutability estimates under the S5F model (20), which describes the relative mutation rate of sequence pentamers during SHM in an independent and separate cohort of healthy subjects. When averaging over pentamers within particular SHM motifs under uniform pentamer frequencies, the S5F model predicts the same ranking as we obtained using the HLP19 model: GYW (mean mutability = 2.46) > WRC (1.87) ∼ WA (1.71) > TW (1.19) > SYC (0.23) ∼ GRS (0.22). The transition/transversion rate ratio (*κ*) estimated by our repertoire-wide model was ∼2, which is also consistent with previous findings (39, 40). Overall, these results show that repertoire-wide parameter estimates obtained using a repertoire-wide phylogenetic approach are broadly consistent with previous expectations in healthy individuals.

### Age Is Associated with Changes in SHM Mutation Biases.

Age and sex are associated with substantial differences in the immune system; for example, older individuals are more vulnerable to infection (41, 42), while females are at a higher risk of developing autoimmune diseases (43). We sought to investigate whether the mutation and selection processes underlying SHM might contribute to these differences.

To investigate potential age- and sex-related differences in SHM targeting, we analyzed the 27 subjects surveyed by Wang et al. (Age dataset; ref. 30), which included both male and female subjects with an age range of 21 to 88 years at the time of sampling. We used multiple linear regression to investigate the effects of age and sex on estimated model parameters. Age and sex were modeled as interaction variables against the estimated substitution rate biases of SHM motifs (i.e., the HLP19 model **h** values; *SI Appendix*, section S1). Because we conducted 20 tests in all (2 dependent and 10 independent variables), we used Benjamini–Hochberg (44) multiple hypothesis test correction to adjust *P* values. Substitution rates in WA (*h*^*WA*^) were significantly negatively associated with age in both male (coefficient = −0.011; adjusted *P* = 0.0012) and female subjects (coefficient = −0.006; adjusted *P* = 0.034). Neither *ω*_*CDR*_ (adjusted slope *P* = 0.58 and 0.76 for males and females, respectively), mean tree length (adjusted slope *P* = 0.58 for males and females), nor any other parameter investigated showed a significant relationship with age in either sex after Benjamini–Hochberg correction (44). These results are consistent with a model in which older individuals have reduced mutation bias toward WA hot spots, possibly reflecting a difference in SHM mechanism in these individuals.

We performed simulation analyses to test whether the observed trends between *h*^*WA*^ and age could be due to biases in our parameter estimation procedure (*Methods* and *SI Appendix*, section S6). For all 20 simulated repetitions of the Age dataset, the *h*^*WA*^ slope coefficients for males and females were closer to zero than their respective empirical estimates (*SI Appendix*, Fig. S6*A*). These results demonstrate that these trends are due to factors other than biases in parameter estimation, given the underlying structure of our datasets and predicted germline sequences.

### Variation in Signatures of Selection Is Uncorrelated with Age, Sex, Epstein–Barr Virus, and Cytomegalovirus Status.

**Fig. 3.**
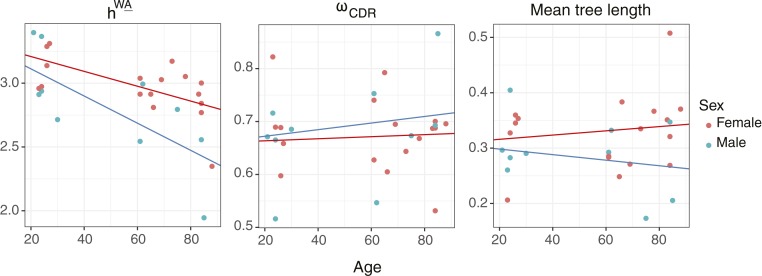
Decrease in estimated WA mutability (*h*^*WA*^) with age. Linear regressions of age against model parameters under the HLP19 model, estimated using ML. Separate regressions are shown for male (blue) and female (red) subjects. The *h*^*WA*^ parameter shows a significant decrease with age in males and females (adjusted slope *P* = 0.0012 and 0.033, respectively).

Antigen-driven selection plays a major role in shaping BCR repertoire diversity. In molecular evolutionary biology, selective dynamics are often characterized by estimating the relative rate of substitutions that change amino acids versus those that do not, often called dN/dS or *ω* (33). Low *ω* values are indicative of fewer amino acid changes than expected, which is generally interpreted as resulting from negative selection. We estimate *ω* separately for the CDRs and FWRs. Estimates of *ω*_*FWR*_ are expected to be lower than those of *ω*_*CDR*_ because FWRs are more structurally constrained than CDRs (45), which are primarily used in antigen binding (1, 21). Consistent with this expectation, we found that in the Age dataset estimated *ω*_*CDR*_ values (range: 0.52 to 0.87, mean: 0.68) were higher than estimated *ω*_*FWR*_ values (range: 0.44 to 0.56, mean: 0.51) in all 27 subjects (*P* < 0.001; paired Wilcoxon test; Fig. 2 and *SI Appendix*, Fig. S10). *ω*_*CDR*_ estimates were also more varied among subjects than *ω*_*FWR*_ values, perhaps representing different individual histories of antigenic stimulation. However, we were unable to find a clear biological correlate of *ω*_*CDR*_ in the Age dataset among the variables provided with the data (30). Specifically, values of *ω*_*CDR*_ did not show a significant relationship with age (slope *P* = 0.66; least squares regression; Fig. 3 and *SI Appendix*, Fig. S10), sex (*P* = 1.0; Wilcoxon rank sum test), Epstein–Barr virus seropositivity (*P* = 0.19; Wilcoxon rank sum test), or cytomegalovirus seropositivity (*P* = 0.19; Wilcoxon rank sum test).

### Postinfluenza Vaccination Repertoires Show Signs of Negative Selection and Longer Tree Length.

Influenza vaccination substantially perturbs the B cell repertoire. A large, antigen-specific plasmablast response is observed in the blood ∼7 d postvaccination which subsides ∼1 wk later (46, 47). To investigate the selective dynamics of this process, we estimated HLP19 substitution model parameters using the repertoires of 3 subjects who were sampled 10 times over the course of influenza vaccination, beginning 8 d prior to vaccination and ending 28 d afterward (31). These subjects otherwise had no other known recent infections or vaccinations. Because we were primarily interested in selection and genetic diversity of these samples, we focused on changes in *ω*_*CDR*_, the relative rate of nonsynonymous/synonymous substitutions, and tree length (the total expected substitutions per codon site within an individual lineage phylogeny).

We found a variety of responses among the subjects. PGP1, the oldest subject of the 3, did not show any clear patterns of change over time, in either mean tree length or *ω*_*CDR*_. Notably, this subject at day +14 had only 141 sequences and consequently very wide 95% CIs, illustrating the importance of correctly estimating model uncertainty in analysis of BCR sequence data.

**Fig. 4.**
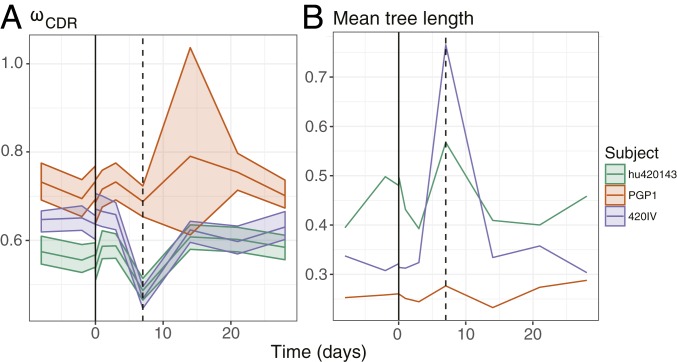
Signatures of selection and diversity following influenza vaccination. (*A*) Estimates of the *ω*_*CDR*_ parameter of the HLP19 model over the course of influenza vaccination. (*B*) Estimates of mean tree length (total substitutions per codon within a lineage, averaged across all lineages within a repertoire) over the course of influenza vaccination. The *x* axis shows the number of days since vaccination; the vertical dashed line represents day 7 postvaccination. The shaded areas in *A* represent the 95% CIs for each parameter estimate, calculated using profile likelihood curves.

In contrast to PGP1, subjects 420IV and hu420143 both showed increased mean tree length at day +7 compared to 1 h prior to vaccination (−1 h), consistent with the expected burst of BCR genetic diversity 7 d postvaccination (46). The estimated mean tree length within a sample was highest at day +7 for subjects 420IV and hu420143, with a fold increase of 2.38 and 1.18 compared to 1 h prior to vaccination (−1 h) (Fig. 4). Consistent with this, multiple large clones in subjects 420IV and hu420143 arose at day +7 (*SI Appendix*, Fig. S8). In addition to increased tree length, day +7 was associated with a significant decrease in *ω*_*CDR*_ in both these subjects (Fig. 4). For 420IV at −1 h, *ω*_*CDR*_ = 0.64 (95% CI: 0.6, 0.66) and at day +7 *ω*_*CDR*_ = 0.47 (95% CI: 0.45, 0.50). For hu420143 at −1 h, *ω*_*CDR*_ = 0.57 (95% CI: 0.54, 0.59) and at day +7 *ω*_*CDR*_ = 0.49 (95% CI: 0.46, 0.51). Interestingly, although 420IV and hu420139 had different prevaccination estimates of *ω*_*CDR*_ (0.64 and 0.57, respectively) their estimates were similar at day +7 (0.47 and 0.49), day +14 (0.62 and 0.61), and day +21 (0.60 and 0.60; Fig. 4). Overall, this indicates that, at the expected date of peak vaccine response, the repertoires of these 2 subjects were characterized by an increase in BCR lineages with large numbers of mutations and signatures of increased negative selection.

We performed simulation analyses to test whether decreased *ω*_*CDR*_ at day +7 in subjects hu420139 and 420IV were due to biases in our parameter estimation procedure (*Methods* and *SI Appendix*, section S6). None of the 20 simulation repetitions performed using the Vaccine dataset was able to reproduce the observed change in *ω*_*CDR*_ at day +7 compared to the prevaccination time point (−1 h; *SI Appendix*, Fig. S6 *B* and *C*), demonstrating that these trends are due to factors besides biases in parameter estimation, given the underlying structure of our datasets and their predicted germline sequences.

### Increased Tree Length Is Associated with Signatures of Negative Selection.

**Fig. 5.**
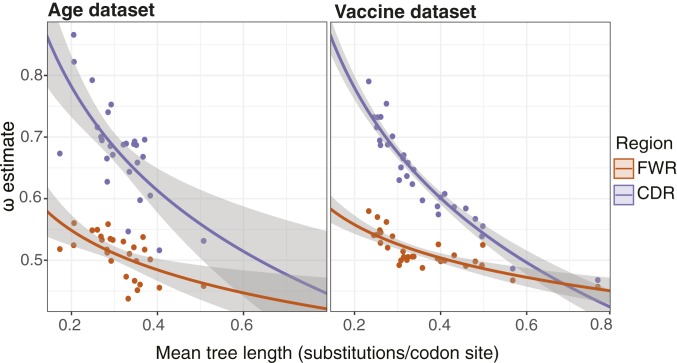
Negative relationship between *ω* and mean tree length. (*Left*) Linear regression between estimates of *ω*_*CDR*_ (purple) and *ω*_*FWR*_ (orange) and the natural log of mean tree length for each subject in the Age dataset. The slope and intercept of *ω*_*CDR*_ against ln(mean tree length) were −0.24 (95% CI = −0.35, −0.14) and 0.39, respectively (*P* < 6 × 10^−5^ for both). The corresponding slope and intercept of *ω*_*FWR*_ were −0.09 (95% CI = −0.14, −0.04) and 0.4 (*P* < 0.002 for both). (*Right*) Linear regression between estimates of *ω*_*CDR*_ (purple) and *ω*_*FWR*_ (orange) and the natural log of mean tree length for each sample in the Vaccine dataset (3 subjects, 10 samples each). The slope and intercept of *ω*_*CDR*_ against ln(mean tree length) were −0.26 (95% CI = −0.29, −0.23) and 0.36, respectively (*P* < 4 × 10^−16^ for both). The corresponding slope and intercept of *ω*_*FWR*_ were −0.08 (95% CI = −0.1, −0.05) and 0.43 (*P* < 4 × 10^−7^ for both). Gray shaded areas in both panels show SE estimates of the log-linear regression.

Our analysis of the Vaccine dataset indicated that, in 2 subjects, there was a concurrent increase in mean tree length and decrease in *ω*_*CDR*_ at day +7 following influenza vaccination. We hypothesized that this relationship between *ω*_*CDR*_ and tree length might be more general and tested this hypothesis using log-linear regression across all 27 subjects of the Age dataset and all 30 samples (10 time points from 3 subjects) of the Vaccine dataset. Across both datasets we observed a consistent and significant negative relationship between both *ω*_*CDR*_ and *ω*_*FWR*_ and mean repertoire tree length (i.e., the average expected substitutions per codon site across all lineages within the repertoire; Fig. 5). This trend was surprisingly similar between datasets, with slopes of linear regressions having overlapping 95% CIs, and was particularly strong in the CDRs. For the Age dataset, the slope of a linear regression of *ω*_*CDR*_ against the ln(mean tree length) was −0.24 (95% CI = −0.35, −0.14; *P* < 6 × 10^−5^), while for the Vaccine dataset the corresponding slope was −0.26 (95% CI = −0.29, −0.23; *P* < 4 × 10^−16^). Overall, these regressions predicted a 32.1% and 41.4% decrease in *ω*_*CDR*_ over the range of mean tree length observed in the Age and Vaccine datasets, respectively. A similar, if weaker, relationship was found between *ω*_*FWR*_ and ln(mean tree length) (Fig. 5; details in legend). This indicates that repertoires with longer lineages (i.e., those with more mutations) are associated with signatures of increased negative selection, particularly in the CDRs.

We performed simulation analyses to test whether the observed trends between *ω* and mean tree length were due to biases in our parameter estimation procedure (*Methods* and *SI Appendix*, section S6). In none of 20 simulations, using both datasets, did we observe a significant relationship between *ω*_*CDR*_ and mean tree length or *ω*_*FWR*_ and mean tree length (*SI Appendix*, Fig. S6 *D* and *E*). However, the simulations in *SI Appendix*, section S6 were performed under a fully context-dependent version of the HLP19 model, which does not completely represent the biased nature of SHM. To test whether a richer model of SHM could potentially reproduce our results, we performed simulations using the S5F model (20), using the tree topologies and branch lengths estimated using maximum parsimony (dnapars v3.679; ref. 48) and the predicted germline sequences of the Age dataset (detailed in *SI Appendix*, section S7). None of 50 such simulation repetitions showed a negative slope between either *ω*_*CDR*_ or *ω*_*FWR*_ and mean tree length as large as that observed for the empirical data (*SI Appendix*, Fig. S7*A*). We therefore conclude that the negative relationship between mean tree length and *ω*_*CDR*_ observed in Fig. 5 is not due simply to inherent biases in our parameter estimation procedure.

## Discussion

Phylogenetic techniques have been used to study B cell lineages for many years (49) and continue to be a powerful tool in understanding affinity maturation (50). Two fundamental issues that arise from the application of phylogenetic techniques to B cell repertoires are 1) the biology underlying B cell affinity maturation violates key assumptions of most phylogenetic models and 2) phylogenetic models are designed typically to work on lineages originating from a common ancestor, but B cell repertoires are composed of multiple lineages with separate ancestries, many of which are composed of only a few unique sequences. If such lineages are each analyzed independently then parameter estimates will be noisy and highly uncertain. Here we introduce a repertoire-wide approach to B cell phylogenetics that addresses these issues. We extend phylogenetic models of SHM evolution so that they are consistent with the known biology of B cell affinity maturation and can share parameters of the sequence evolution process across all lineages within a repertoire. This approach outperforms the alternative of averaging parameters estimated for each lineage individually and provides a principled framework for testing evolutionary hypotheses about mutation and selection in B cell repertoires. By applying our approach to empirical data we find evidence consistent with dysregulation of SHM in older subjects, increased signatures of negative selection associated with influenza vaccine response, and a relationship between mean tree lengths and signatures of negative selection. Our methods are implemented in the program IgPhyML (https://igphyml.readthedocs.io).

We first used our repertoire-wide framework to demonstrate a negative association between age and the estimated mutability of WA hot-spot motifs in males and females. Previous studies have shown that aging is associated with a decrease in the affinity, specificity, and diversity of antibodies produced (51–54), as well as with a number of changes at the repertoire level, including longer CDR3s, higher levels of SHM, and persistent clonal lineages in the blood (30). It is possible that age-related dysregulation of SHM machinery plays a role in phenomena associated with immunosenescence. Our finding that older individuals tend to have altered mutability of WA motifs is consistent with this hypothesis. SHM at A/T sites is thought to be driven by error-prone DNA polymerase η (55), so dysregulation of pathways relating to this protein could form a basis for this pattern. No significant relationship was found between age and any other variable investigated, including *ω*_*CDR*_ and mean tree length (Fig. 3). This is consistent with a recent large study that found no difference between young and elderly subjects in either mean total mutation frequency or the ratio of nonsynonymous-to-synonymous mutations (56). While statistically significant, the negative relationship we observed between WA mutability and age was modest in size and is based on a single cohort. It is possible this trend is driven by other confounding factors we have not considered, and future analyses with more subjects will be needed to validate this trend generally.

We further used our repertoire-wide phylogenetic approach to characterize BCR molecular evolution during vaccination. BCRs during affinity maturation are subject to multiple selective pressures: positive selection to introduce new affinity-increasing amino acid variants (higher *ω*) and negative selection to remove affinity-decreasing variants (lower *ω*). The balance of these forces, and therefore the overall *ω*, may vary over time. Mutational fitnesses are often visualized as a fitness “landscape” in which the sequence space surrounding optimal variants is represented as a “peak” (57). A population ascends a peak by accumulating advantageous mutations and avoids descending by removing deleterious mutations through negative selection. However, as the population nears the summit, the proportion of replacement mutations that can increase fitness declines, hence overall *ω* lowers. A priori, we might expect positive selection to predominate during vaccine response. However, in our analysis we found that lineages present at the time of peak influenza vaccine response show signs of decreased positive selection (lower *ω*) on CDRs. We suggest this is because B cell lineages with a history of affinity maturation during influenza infection or vaccination will likely have already evolved effective or nearly effective neutralization at the time of vaccination [no subject was naïve to the vaccine antigens (31)], resulting in a greater proportion of amino acid changes being deleterious or neutral (58). This would result in lower *ω*_*CDR*_ values. This effect may be particularly marked during influenza vaccine response because B cells activated by influenza vaccination in adults are expected to derive from reactivated memory B cell lineages (59), depending on the extent to which subjects have been previously exposed to the epitopes in the vaccine (60).

We also observed a negative relationship between tree length and *ω*_*CDR*_ across both of our datasets (Fig. 5). This relationship is remarkably consistent given that our combined datasets contain a total of 30 subjects of different age, sex, and treatment status. None of the simulation analyses performed under a null model were able to reproduce this result (*SI Appendix*, sections S6 and S7), so this relationship is unlikely to be due to a bias or intrinsic correlation between these variables in our estimation procedure. In the absence of other obvious confounding factors, we posit a simple biological explanation: As B cell clones accumulate mutations through repeated rounds of affinity maturation, their binding affinity to target antigen increases and consequently the benefit of random amino acid changes (i.e., new mutations) decreases (58). This idea that the rate of fitness-increasing mutations decreases is a straightforward implication of a population nearing a “peak” within a fitness landscape detailed earlier (57). Sheng et al. (27) demonstrated evidence of this process (which they termed the “affinity maturation selection” model) in anti-HIV broadly neutralizing antibody (bnAb) lineages. This explanation is also consistent with the findings of Yaari et al. (61) showing that mutations earlier in B cell lineage trees from healthy subjects show clearer signs of positive selection than more recent mutations. Our results suggest that decreased rates of nonsynonymous mutations relative to synonymous mutations, as observed in HIV bnAb lineages (27), BCR repertoires during vaccine response (Figs. 4 and 5), and even in healthy subjects with no obvious signs of infection (Fig. 5 and ref. 61), are all special cases of a general feature of affinity maturation.

While the repertoire-wide phylogenetic method introduced here has several advantages over previous approaches, other techniques for characterizing selection and SHM in BCR sequences are available, and ultimately the most appropriate approach will depend on the hypothesis and data being tested. For quantifying selection pressure in B cell lineages, one popular approach is BASELINe (21), a nonphylogenetic method that characterizes selection pressure by detecting an excess (or lack) of replacement to silent mutations (R/S) between a sequence and its predicted germline. Unlike phylogenetic techniques, BASELINe represents clonal lineages using single representative sequences. This may lower statistical power and, depending on the technique used to generate the representative sequence, may bias inference of selection. Representing a clone using a single sequence also has the disadvantages that 1) codons with multiple mutations may be ignored and 2) all mutations are assessed in the sequence context of their predicted germline sequence rather than an immediate ancestor. Both of these issues are dealt with naturally in our phylogenetic framework. McCoy et al. (62) used a Bayesian regularization technique to derive site-wise estimates of *ω*, giving a more finely resolved interpretation. Like BASELINe, their technique used individual sequences paired with predicted germline ancestors, rather than modeling phylogenetic lineage structure, and likely has similar limitations. In some cases the biased nature of SHM motifs, rather than selection, is the primary interest (20, 40). To characterize SHM in this study we used previously defined SHM motifs marginalized over codon boundaries using a mean field approximation. By contrast, Feng et al. (63) used a survival analysis framework to infer SHM motifs de novo from individual sequence datasets. That method, however, did not estimate *ω* so was limited to studying biased mutation motifs. In summary, we argue that the field will be best served by having access to a variety of methods with nonoverlapping assumptions that can best characterize different aspects of the complex affinity maturation process.

One drawback in using phylogenetic models to characterize B cell lineage evolution is that the relationship between the strength of selection and within-population estimates of *ω* can vary by timescale (64), which can make interpretation of estimates difficult. However, the issues outlined in ref. 64 are less of a problem for B cell lineages because their ancestral (i.e., germline) states are knowable a priori, which makes it possible to distinguish between conserved ancestral positions and fixed derived sites resulting from selective sweeps. Computational complexity can be a significant limitation when using phylogenetic parameter estimation in a repertoire-wide framework. The method becomes increasingly impractical with more than a few thousand sequences, necessitating subsampling of larger datasets. Perhaps the most obvious disadvantage of repertoire-wide parameter estimation is that by constraining parameter values so that they are identical for all lineages within a repertoire we mask any potential parameter variation among lineages. Thus, repertoire-wide estimates should not be used to make statements about individual lineages. However, our proposed framework easily accommodates the possibility of designating some parameters whose values could be estimated for each lineage individually as would be done in a more general hierarchical model (23). This may be useful for parameters such as *ω*_*CDR*_, which might reflect lineage-specific histories of antigen-driven selection (17). As an example of this approach, we explored heterogeneity in estimates of *ω*_*CDR*_ among lineages of different sizes for one repertoire (*SI Appendix*, section S9). This analysis revealed significantly lower *ω*_*CDR*_ in groups of larger clones, compared to smaller clones within the same repertoire. It is unclear whether estimation of individual *κ* and **h** values would yield useful insights, since these parameters relate primarily to biases resulting from SHM, and there is little a priori reason to believe they might vary among B cell lineages within an individual. However, it is clear that estimating parameters (e.g., *ω*_*CDR*_) for each lineage individually will lead to issues with overfitting (e.g., when all CDR mutations within a lineage are nonsynonymous). Further work will be needed to resolve lineage heterogeneity within individual repertoires.

A repertoire-wide phylogenetic approach to BCR phylogenetics is justified theoretically and provides a principled statistical framework for the analysis of B cell repertoires. Our methods are implemented in the program IgPhyML (v1.0.7; https://igphyml.readthedocs.io), which is freely available and integrated into the Immcantation suite (http://immcantation.org).

All primary data used in this study was previously made publicly available through ref. 30 (Age dataset) and ref. 65 (Vaccine dataset), which resequenced samples from ref. 31. Scripts used to generate simulated datasets and perform analyses are available at Zenodo (https://doi.org/10.5281/zenodo.3479844) (66).

## Supplementary Material

Supplementary File
