# Supplementary material for: Repertoire-wide phylogenetic models of B cell molecular evolution reveal evolutionary signatures of aging and vaccination
Source: Proc Natl Acad Sci U S A. 2019 Oct 21;116(45):22664–72. doi: 10.1073/pnas.1906020116 (PMC6842591; doi:10.1073/pnas.1906020116)
Supplement: Supplementary File [file pnas.1906020116.sapp.pdf]

PNAS
www.pnas.org

Supplementary Information for

Repertoire-wide phylogenetic models of B cell molecular evolution reveal evolutionary signatures of aging and vaccination

Kenneth B. Hoehn, Jason A. Vander Heiden, Julian Q. Zhou, Gerton Lunter, Oliver G. Pybus, Steven H. Kleinstein

To whom correspondence should be addressed:

Prof. Steven Kleinstein
Email: steven.kleinstein@yale.edu

Prof. Oliver Pybus
Email: oliver.pybus@zoo.ox.ac.uk

This PDF file includes:

Sections S1-10

Section S1: Supplemental Methods

HLP19 model definition

We begin with the HLP17 substitution model (1), in which instantaneous rates of codon substitution are parameterized by the nonsynonymous/synonymous mutation rate ratio (ω), transition/transversion mutation rate ratio (κ), a vector of codon frequencies ($\boldsymbol{\pi}$), and a vector of modified substitution rates $\mathbf{h} = (h^{WR\underline{C}}, h^{\underline{G}YW} \ldots, h^{(a)})$ where a is an SHM hot- or cold-spot motif, such as WR<u>C</u> (2); W=A/T, R=A/G). We modify the HLP17 model by removing codon frequencies from the **Q** matrix, and parameterize the instantaneous rate of change from codon i to j, where $i \neq j$, as:

$$q_{ij} = \begin{cases} 0 & i \to j \text{ more than } 1 \text{ nucleotide change} \\ 1 + \sum_a b_{ij}^{(a)} h^{(a)} & i \to j \text{ synonymous transversion} \\ \kappa\left(1 + \sum_a b_{ij}^{(a)} h^{(a)}\right) & i \to j \text{ synonymous transition} \\ \omega\left(1 + \sum_a b_{ij}^{(a)} h^{(a)}\right) & i \to j \text{ nonsynonymous transversion} \\ \omega\kappa\left(1 + \sum_a b_{ij}^{(a)} h^{(a)}\right) & i \to j \text{ nonsynonymous transition} \end{cases}$$

where $b_{ij}^{(a)}$ is the probability that a mutation from i to j involves the underlined base in motif a (e.g. WR<u>C</u>, where W = A/T, R = A/G, and C experiences an altered substitution rate). The values

of $b_{ij}^{(a)}$ are calculated by marginalizing over all possible 5' and 3' flanking sense codons as follows:

$$b_{ij}^{(a)} = \sum_{k=1}^{61}\sum_{g=1}^{61} \pi_k \, \pi_g I(i,j,k,g,a)$$

where $I(i,j,k,g,a)$ is the indicator function:

$$I(i,j,k,g,a) = \begin{cases} 1 & kig \rightarrow kjg \ is\ a\ mutation\ from\ motif \text{ a} \\ 0 & otherwise \end{cases}$$

Importantly, although the $\boldsymbol{\pi}$ values are not used explicitly in the definition of the substitution matrix, **Q** does depend on $\boldsymbol{\pi}$ through $\mathbf{B}^{(a)} = b_{ij}^{(a)}$ and through matrix calibration (see next section). Diagonal elements of the **Q** matrix (i.e. $i = j$) are equal to the negative sum of the off-diagonal elements of the same row. More explicitly, for each codon i:

$$q_{ii} = -\sum_{j=1, j\neq i}^{61} q_{ij}$$

This ensures that the rows of the **Q** matrix sum to zero.

Rate matrix calibration

Q matrices are typically calibrated using equilibrium frequencies such that branch lengths are equal to the expected number of substitutions per site (3). Namely, the expected number of substitutions per site and per unit of time (μ) at equilibrium for a **Q** matrix is calculated as:

$$\mu = -\sum_{i=1}^{61} \pi_i q_{ii}$$

Each element in the **Q** matrix is then divided by μ, such that for the resulting calibrated **Q** matrix, $\mu = 1$. This calibration makes phylogenetic branch lengths interpretable as the expected number of substitutions per site, assuming codon frequencies have converged to a stationary distribution. Codon frequencies ($\boldsymbol{\pi}$) are an important part of this calculation, and if they do not accurately reflect the composition of the sequence at a particular time point this may lead to inaccurate branch length estimates. Typically, codon frequencies are assumed to have reached their stationary distribution, and $\boldsymbol{\pi}$ is set to the equilibrium codon frequency distribution. This assumption is made in virtually all substitution models, including HLP17 (1), which estimates state frequencies using maximum likelihood.

Calibrating **Q** matrices using equilibrium frequencies is likely a poor choice for B cell lineages because B cells are known to begin out of sequence equilibrium and change over time (4). Because the germline sequence of B cell lineages is predicted or specified before phylogenetic estimation, it is possible to use either the empirical codon frequencies of the germline sequence ($\boldsymbol{\pi}^{(g)}$) or the empirical codon frequencies of the tip sequences ($\boldsymbol{\pi}^{(t)}$) to calibrate the **Q** matrix. However, both of these reflect codon frequencies at only the beginning and end of the affinity maturation evolutionary process. Because codon frequencies are modelled as changing from $\boldsymbol{\pi}^{(g)}$ to $\boldsymbol{\pi}^{(t)}$ using the substitution process described in **Q**, we can use $\boldsymbol{\pi}^{(g)}$ and **Q** to predict the codon frequency change over a defined interval. We use this to calibrate our **Q** matrix using predicted codon frequencies at the midpoint of the tree ($\boldsymbol{\pi}^{(m)}$). To predict these values, we first calibrate the **Q** matrix using $\boldsymbol{\pi}^{(g)}$ to give $\mathbf{Q}^{(g)}$ and calculate the transition probability matrix $\mathbf{P}^{(m)} = \exp(\mathbf{Q}^{(g)}m)$, where m is half the mean root-tip divergence of all sequences in the initial tree –

which we estimate using the GY94 (5, 6) model on the same data. From these pieces of information, we calculate $\boldsymbol{\pi}^{(\mathrm{m})}$ using:

$$\pi_i^{(\mathrm{m})} = \sum_j \pi_j^{(\mathrm{g})} P_{ji}^{(\mathrm{m})}$$

and use these values to calibrate $\mathbf{Q}$ to give $\mathbf{Q}^{(\mathrm{m})}$, which is scaled such that branch lengths are equal to the expected number of codon substitutions per site at the midpoint of the starting tree, and is used for all likelihood calculations. Intuitively, $\pi_j^{(\mathrm{g})} P_{ji}^{(\mathrm{m})}$ is the probability that the Markov chain begins in codon *j* at the germline and is in codon *i* after interval *m* has elapsed. In this way, $\pi_i^{(\mathrm{m})}$ is computed by marginalizing over all possible ways the Markov chain could be in codon *i* after interval *m* given the starting frequencies $\boldsymbol{\pi}^{(\mathrm{g})}$. This gives the predicted frequency of codons over an interval equal to the average midpoint between the root and each tip. Because this procedure does not use codon frequencies from the tips, the accuracy of the predicted midpoint frequencies depends on how well the substitution process is described by the $\mathbf{Q}$ matrix.

In addition to being used in the calibration of branch lengths, codon frequencies are also used to calculate $b_{ij}^{(a)}$ values. To solve for the latter, we use $\boldsymbol{\pi}^{(\mathrm{g})}$ values to create the $\mathbf{B}^{(a)}$ matrices used in constructing $\mathbf{Q}^{(\mathrm{g})}$. Ideally, we would then use the estimated $\boldsymbol{\pi}^{(\mathrm{m})}$ values to construct the matrices $\mathbf{B}^{(a)}$ used in $\mathbf{Q}^{(\mathrm{m})}$. However, doing so would require recalculating all $b_{ij}^{(a)}$ values each time any parameter is updated. This is computationally expensive, requiring 61^4 computations rather than 61^2 computations for simply updating the $\mathbf{Q}$ matrix. We therefore calculate each element of $b_{ij}^{(a)}$ once using the values of $\boldsymbol{\pi}^{(\mathrm{g})}$ and keep the $\mathbf{B}^{(a)}$ matrices constant throughout remaining parameter estimation. This dramatically improves runtime. In this way, $\mathbf{Q}^{(\mathrm{m})}$ depends on $\boldsymbol{\pi}^{(\mathrm{m})}$ through calibration and on $\boldsymbol{\pi}^{(\mathrm{g})}$ through $\mathbf{B}^{(a)}$ matrix values.

Further, in many of our analyses, we partition our sequences into complementarity determining regions (CDRs) and framework regions (FWRs), and estimate separate values of ω for each. In these cases, $\boldsymbol{\pi}^{(\mathrm{g})}$ and $\boldsymbol{\pi}^{(\mathrm{m})}$ estimation is the same as described above but performed on the separate partitions independently: each tree has two $\mathbf{Q}$ matrices, which are identical except that $\mathbf{Q}_{\mathrm{CDR}}^{(\mathrm{m})}$ uses ω_{CDR} and is calibrated using codon frequencies in the CDRs, while $\mathbf{Q}_{\mathrm{FWR}}^{(\mathrm{m})}$ uses ω_{FWR} and is calibrated using codon frequencies in the FWRs. Likelihood calculations involving ambiguous codons in the root sequence are averaged over all compatible codons, weighted by their frequencies in either CDRs or FWRs obtained from an empirical dataset of four healthy subjects (7).

Overall, compared to HLP17, the updated model defined above, which we refer to as HLP19, uses less than half the number of free parameters and models the non-equilibrium nature of affinity maturation in a more interpretable manner. It is also more formally similar to previous nonreversible phylogenetic model forms (8, 9).

Repertoire dataset processing

The “Age” dataset consists of samples taken from 27 healthy individuals in two consecutive years (10). Subjects varied in age from 20 to 81 years old, and both male and female subjects were included. Sequencing was performed in three replicates: two using genomic DNA and one using mRNA. Raw bulk-sequencing reads using the 454 (Roche) platform from PBMCs as described in (10) were preprocessed using pRESTO v0.4.1. Briefly, reads were quality controlled

by filtering reads with length shorter than 250 nucleotides and those with a mean Phred quality score below 20. Reads were aligned against V and C region primers, allowing for a maximum error rate of 0.2. Duplicate reads and reads that were observed only once (singletons) were removed. Following preprocessing, V(D)J assignment was performed using IgBLAST v1.7.0 (11) against the IMGT human germline reference database (IMGT/GENE-DB v3.1.16; retrieved June 19th, 2017) (12). Default IgBLAST parameters were used. Postprocessing of IgBLAST output and clonal clustering were performed using Change-O v0.3.7 (13). Putative non-productively rearranged sequences were filtered. To infer clonal lineages, sequences were first partitioned based on common IGHV gene annotation, IGHJ gene annotation, and junction region length. IGHV and IGHJ gene annotations for each partition were determined by the union of ambiguous assignments within each junction length partition having at least one overlapping gene annotation among all gene assignments. Within these larger groups, sequences differing from one another by a normalized Hamming distance of 0.1 within the junction region were clustered as clones via single-linkage hierarchical clustering (14). The clonal distance threshold was determined by manual inspection to identify the local minima between the two modes of the bimodal distance-to-nearest histogram produced using SHazaM v0.1.8)(15). The V and J genes of unmutated germline ancestors of each clone were predicted using Change-O, with D segment and N/P regions masked by "N" nucleotides. Clonal clustering was performed using all three sequencing replicates (two obtained from genomic DNA and one mRNA) pooled together; however, after this mRNA sequences were separated and exclusively used for subsequent analysis.

The “Vaccine” dataset consists of samples from three male donors at 10 time points: -8 days, -2 days, -1 hour, +1 hour, +1 day, +3 days, +7 days, +14 days, and +28 days relative to seasonal influenza vaccination (16). The sample collection protocol is available in (16); sequencing, as well as data preprocessing for read quality and inference of clonal clusters is described in (14). Briefly, samples were re-sequenced from mRNA using 5’RACE with unique molecular identifiers (UMIs) and sequenced using the Illumina MiSeq platform. Using pRESTO v0.4 (17), low quality bases in reads (Phred score < 5) were masked with Ns, paired-end reads were matched and assembled, and assembled sequences with the same UMI were collapsed into a consensus sequence (14). Following preprocessing, V(D)J assignment was performed using IgBLAST v1.4.0 (11) against the IMGT human germline reference database (IMGT/GENE-DB v3.1.16; retrieved June 19th, 2017) (12). Default IgBLAST parameters were used. Putative non-productively rearranged sequences were filtered. As with the Age dataset, clonal lineages were inferred by grouping sequences based on common IGHV gene, IGHJ gene, and junction length. Within these groups, sequences differing from one another by a normalized Hamming distance of 0.1 within the junction region were clustered into clones via single-linkage hierarchical clustering (14). The V and J genes of the unmutated germline ancestors for each clone were predicted using Change-O v0.4.3 (13), with D segment and N/P regions masked by ‘N’ nucleotides.

For HLP17/19 phylogenetic analysis it is necessary for all sequences within a particular clone to be placed in a codon alignment with the correct reading frame. To achieve this, sequences from each clone were multiple aligned using the IMGT numbering scheme, which was also used to determine the locations of CDRs and FWRs (18). It is possible that BCRs will accumulate

insertion mutations relative to their germline precursor over the course of affinity maturation. In this case, Change-O removes insertions in its IMGT sequence alignment in order to preserve numbering. However, frame-preserving insertions occasionally occur mid-codon. Removing these insertions brings together nucleotides that do not actually form an observed codon in the sequence, creating a potentially false codon substitution. We detected these events by comparing input sequences to IMGT aligned sequences in Change-O files, and masking them using "N" nucleotides. We then trimmed all gap-only columns in IMGT alignments within each clone. This functionality, as well as converting Change-O files to IgPhyML inputs, is now included in the BuildTrees tool as part of Change-O v0.4.3+ (13).

Sequences from both years within the same patient were pooled together in the Age dataset, resulting in a single repertoire sample per subject. From both datasets, lineages with fewer than 2 unique sequences (not including the predicted germline) were discarded. Due to the large number of sequences per sample in the Vaccine dataset, and the computational complexity of our parameter estimation procedure, these repertoires were subsampled at each subject and time point combination. Sequences from each sample were subsampled without replacement until either the sample contained at least 3,000 unique sequences within clones of at least 2 unique sequences (occasionally this resulted in some datasets having up to 3,001 sequences), or all sequences were used. This cutoff was chosen because all samples from subject hu420139 and 420IV, and 5/10 samples from PGP1 had >3000 unique sequences in non-singleton clones.

Section S2: Simulating under the HLP19 model

We performed multiple simulation analyses to validate our parameter estimation procedure under a fully context-dependent version of the HLP19 model (**Sections S3**, **S4**, and **S6**). These were performed under different parameter values, tree topologies, and initial sequences as detailed in their respective files, but they share a common means of simulating sequence changes along a given tree. These processes require a starting tree topology, branch lengths, a germline sequence, and substitution parameters to be specified in advance. For a given lineage tree, the starting germline progenitor sequence is specified as either a random naïve B cell sequence (**Sections S3** and **S4**) or the previously identified germline sequence in the empirical data (see **Section S6**). For each branch in the tree we simulated the number of nucleotide substitutions by drawing from a Poisson distribution whose mean equals the branch length divided by three and multiplied by the number of codons in the germline sequence. Branch lengths were divided by three because branches lengths estimated under all substitution models used in this paper are in units of expected codon substitutions per site, while the simulation procedure operates on single nucleotides. For a given sequence we then calculated the probability of all single substitutions under the HLP19 **Q** matrix (**Section S1**) based on their full nucleotide context (rather than the mean field approximation; described by parameters $h^{WR\underline{C}}$, $h^{\underline{G}YW}$, $h^{W\underline{A}}$, $h^{\underline{T}W}$, $h^{SY\underline{C}}$, and $h^{\underline{G}RS}$), whether the substitution was synonymous or non-synonymous (described by parameters ω_{FWR} and ω_{CDR} depending on whether the site was in the FWRs or CDRs), and whether the substitution was a transition or transversion (described by κ). This process began at the specified germline progenitor and continued down the tree to the tips. We then masked the CDR3 of the germline sequence to mimic the behavior of the CreateGermlines prediction program, which is unable to

reliably predict germline D segment and N/P regions (13). We also removed the CDR3 regions of all sequences in each dataset to avoid potential issues generated with our inability to properly predict this region. This process repeated for all clonal lineage trees in a dataset. These simulations were performed using custom Perl scripts, which are available at https://bitbucket.org/kleinstein/projects.

Issues with the simulation approach in Hoehn et al 2017

Hoehn *et al.* (1) used a similar approach to simulate sequences under the HLP17 substitution model. However, the simulations in Hoehn *et al.* (1) differ from the approach described above; specifically, in Hoehn *et al*. (1) the relative substitution probabilities for each site were computed by calibrating the **Q** matrix of the HLP17 model with its codon frequencies so that branch lengths could be interpreted as substitutions per site (discussed in **Section S1**), and then exponentiating this matrix to give the **P** matrix of substitution probabilities for each codon. Codon substitutions were then introduced based on these probabilities at each site. However, because **Q** matrices are calibrated using codon frequencies, the degree to which branch lengths can be accurately interpreted as substitutions per site depends on how accurately the codon frequencies used reflect the composition of the sequences being simulated. For the HLP17 model, these frequencies are estimated through maximum likelihood as free parameters – they are not necessarily tied to the composition of the sequences. This results in branch lengths that less accurately reflect the expected substitutions per site than standard models (this inaccuracy is confirmed in **Section S3**). Unfortunately, because the simulations performed in that paper used calibrated, exponentiated **Q** matrices, this inaccuracy was present in the simulated data itself,

leading to simulated datasets with fewer substitutions than expected given their branch lengths. This resulted in the HLP17 model outperforming the GY94 model in branch length estimation when compared to the original branch lengths of the trees used in simulations. While an accurate description of the simulation procedure used, this result is likely specific to this particular simulation approach and not general, and does not hold up in the fairer trials presented in this paper. The approach taken here (outlined above) avoids this issue by simply weighting all possible single nucleotide substitutions for all sites in a sequence by their values in the substitution matrix, randomly choosing a substitution, and then repeating this process on the new sequence for a given number of substitutions. This simulation process makes no explicit assumptions regarding codon frequencies – they change as a result of the substitution parameters, starting sequence, and lineage tree length. Further, the simulation analyses used in this paper explicitly define the number of nucleotide substitutions added on each branch. This means branch length estimates in the analyses presented in this paper (**Section S3**) are scored for accuracy based on the actual number of substitutions per codon site used in the simulation procedure, rather than the branch lengths of the original tree topology as done in Hoehn *et al.* (1).

Section S3: Performance of GY94, HLP17, and HLP19 substitution models

The goal of this simulation analysis is to compare the parameter estimation performance of the GY94 (5, 6), HLP17 (1), and HLP19 substitution models. Simulations were performed on two trees with identical topologies and total tree length (i.e. expected number of substitutions), but different branch length distributions (*split* and *long*; see **Figure S3a** below). We created artificial datasets by simulating sequences using 200 copies of either *split* or *long* tree topologies. Simulations were performed as described in **Section S2**. In these analyses, we selected a random naïve heavy chain sequence from a dataset of two healthy individuals (7) for each tree in each dataset. We further used two sets of substitution parameters. In the first, named *hot*, $\kappa = 2$, $\omega_{FWR} = 0.4$, $\omega_{CDR} = 0.7$, $h^{WR\underline{C}} = 2$, $h^{\underline{G}YW} = 3$, $h^{W\underline{A}} = 2$, $h^{\underline{T}W} = 1$, $h^{SY\underline{C}} = -0.6$, and $h^{\underline{G}RS} = -0.6$. In the second, *hotter*, $\kappa = 2$, $\omega_{FWR} = 0.4$, $\omega_{CDR} = 0.7$, $h^{WR\underline{C}} = 4$, $h^{\underline{G}YW} = 6$, $h^{W\underline{A}} = 4$, $h^{\underline{T}W} = 2$, $h^{SY\underline{C}} = -0.6$, and $h^{\underline{G}RS} = -0.6$. Overall, we created 50 datasets for each combination of tree topology and substitution model.

To compare model performance, for each simulated dataset we used maximum likelihood to estimate tree topology, branch lengths, κ, ω_{FWR}, and ω_{CDR} the GY94, HLP17 and HLP19 models. We also estimated $h^{WR\underline{C}}$, $h^{\underline{G}YW}$, $h^{W\underline{A}}$, $h^{\underline{T}W}$, $h^{SY\underline{C}}$, and $h^{\underline{G}RS}$ for HLP17 and HLP19. All of these parameters were estimated at the repertoire level. Codon frequencies ($\boldsymbol{\pi}$) shared among all lineages within the repertoire were estimated using maximum likelihood for the HLP17 model, as empirical frequencies within each lineage (pooled across FWR and CDRs) for GY94, and using the procedure detailed in **Section S1** for HLP19. The CDR3 region was removed for all sequences after simulation. For each parameter, we calculated bias as the mean (true–

estimated)/true value across all 50 datasets of each type. True branch lengths for each simulated lineage were defined as the number of substitutions that occurred outside of the CDR3 along a particular branch, divided by the number of codon sites outside of the CDR3 in the simulated alignment. The results of these tests are shown in **Figures S3b** and **S3c** below.

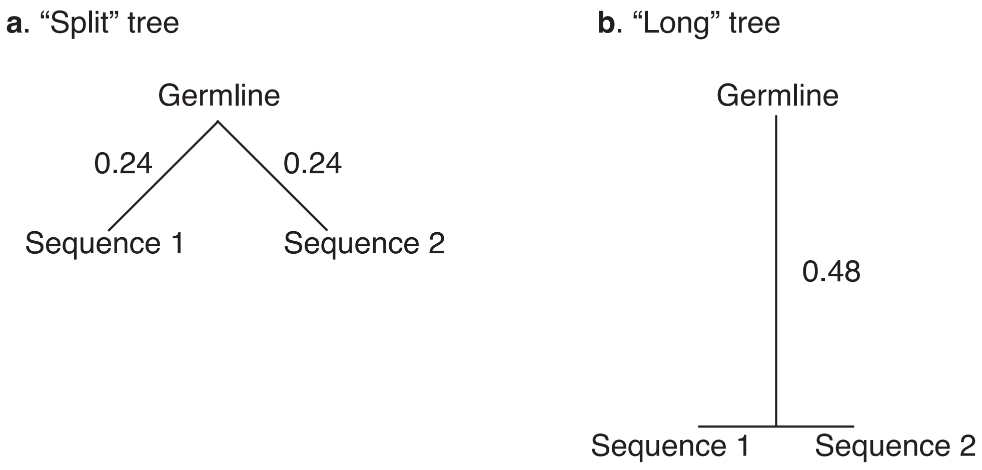

Figure S3a: Trees used in simulation analyses described in **Section S3**. Repertoires specified as "split" used the tree in **a**, and repertoires specified as "long" used the tree in **b**. Branch lengths are in codon substitutions per site. All branches with unlabeled lengths are equal to zero. "Germline" is the germline progenitor sequence of the lineage, while Sequence 1 and Sequence 2 are the data sequences produced by the simulation process.

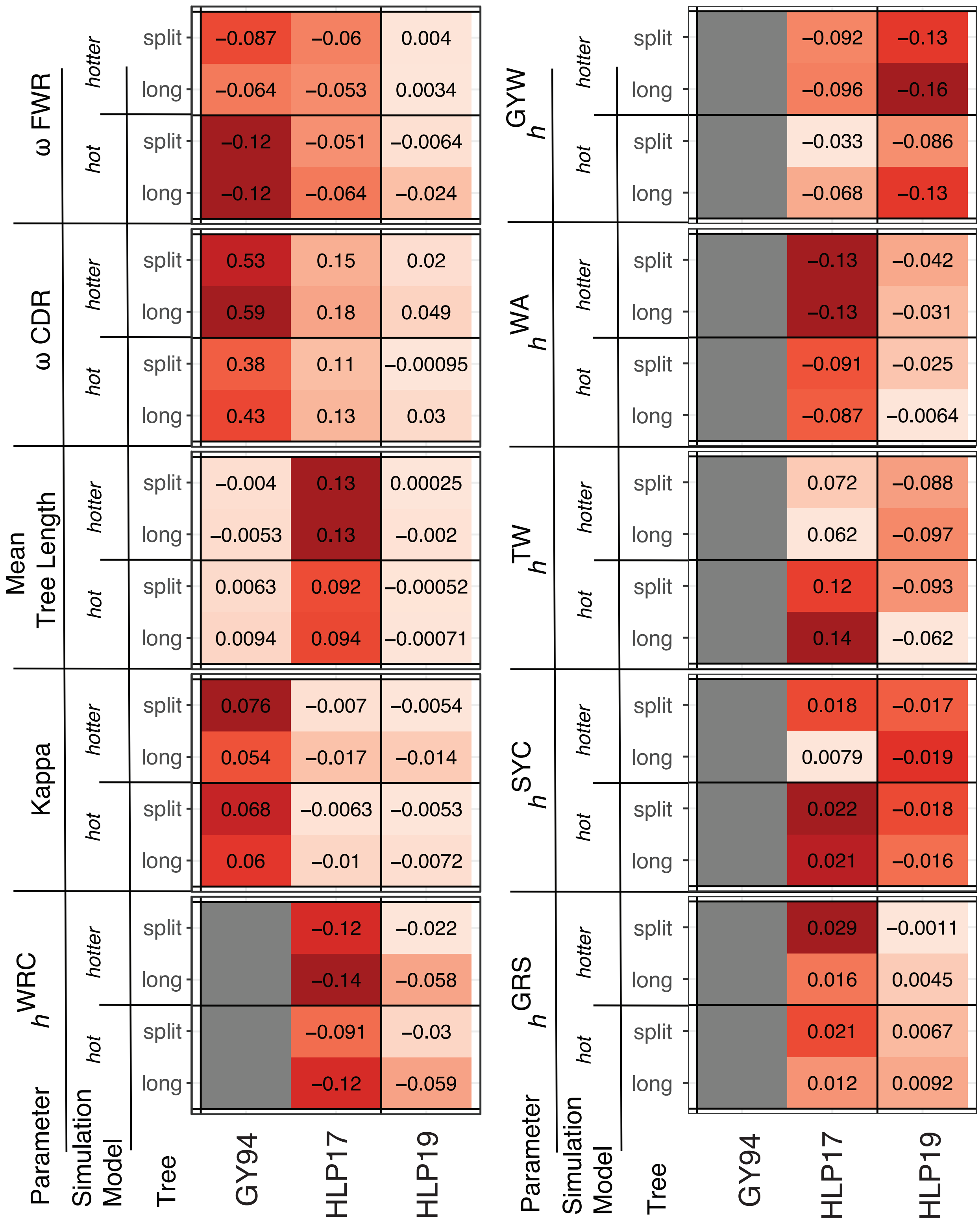

Figure S3b: Mean bias in parameter estimation for different substitution models. Y axis labels show the parameter estimated, as well as the tree type and model used in simulation. X axis labels show the substitution model used to estimate parameters. See **Section S3** for full details.

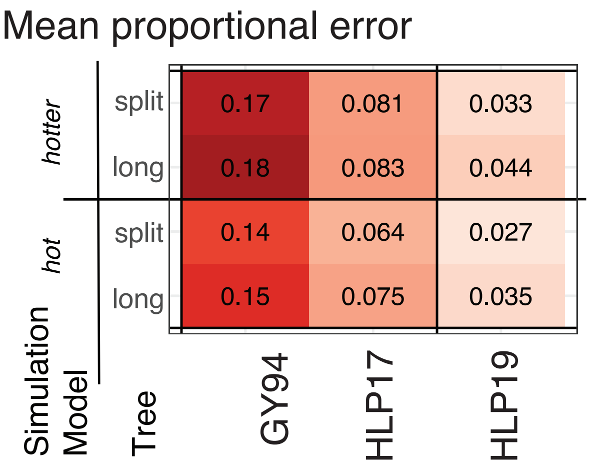

Figure S3c: Mean absolute bias in substitution parameter estimation for different substitution models. Mean error was calculated as the mean absolute value all parameters shown in **Figure S3b**. **h** values were not estimated for GY94, so were not included in this calculation.

Section S4: Performance of individual versus repertoire-wide model parameter estimation

The goal of this simulation analysis was to quantify the performance of parameter estimation using the repertoire-wide phylogenetic approach vs. individual lineages using the HLP19 substitution model. We used lineage trees from a single repertoire (subject 97 in the Age dataset) which was chosen due to its greater number of large lineages compared to other samples in the Age dataset. As described in **Methods**: *Phylogenetic model parameter and topology estimation*, we first estimated maximum likelihood tree topologies, branch lengths, and shared values of ω, and κ for all lineages within each repertoire under the GY94 model. Codon base frequencies were set to their empirical estimates across all lineages within the repertoire using a CF3X4 model (19). As in **Section S3**, we selected a random heavy chain sequence from a dataset of naïve B cells from two healthy individuals (7) to act as the germline sequence for each tree in each dataset. The CDR3 region was removed for all sequences after simulation. Simulations were then performed using the procedure outlined in **Section S2**, using substitution parameters $\kappa = 2$, $h^{WR\underline{C}} = 4$, $h^{\underline{G}YW} = 6$, $h^{W\underline{A}} = 3$, $h^{\underline{T}W} = 1$, $h^{SY\underline{C}} = -0.6$, and $h^{\underline{G}RS} = -0.6$. These were chosen to be similar to those estimated from the Age dataset (**Figure 2**). We then repeated the parameter estimation procedure in **Methods**: *Phylogenetic model parameter and topology estimation* by (briefly) first estimating tree topologies, branch lengths, and values of ω, and κ under the GY94 model, and then estimating substitution parameters under the HLP19 model. For GY94 estimations, codon base frequencies were set to their empirical estimates across all lineages within the repertoire using a CF3X4 model (19). We repeated this process both for each lineage individually, and for each repertoire using a repertoire-wide model. For individual trees, all parameters were estimated for each individual lineage.

Two types of simulations were performed. In the first simulation, parameters were identical among lineages, and $\omega_{FWR} = 0.5$, $\omega_{CDR} = 0.7$. We compared the accuracy of parameter estimates at the repertoire level versus the mean parameters of individual lineages containing at least 2, 10, and 30 sequences (**Table S4b**). In the second simulation, the κ and **h** parameters were identical among lineages, but ω parameters were allowed to vary. This is motivated by the expectation that the strength of selection may vary among lineages but the mutational biases of SHM (i.e. hot-spots and cold-spots) are expected to be constant. For each lineage, the κ and **h** parameters were identical to previous simulations, but ω_{CDR} was drawn from a gamma (shape=10, scale=0.07) distribution (mean = 0.7), and ω_{FWR} was drawn from a gamma (shape=10, scale=0.05) distribution (mean = 0.5). These are shown in **Figure S4a**. The results of these simulation analyses are shown in **Table S4c**. We performed 50 repetitions of each type of simulation.

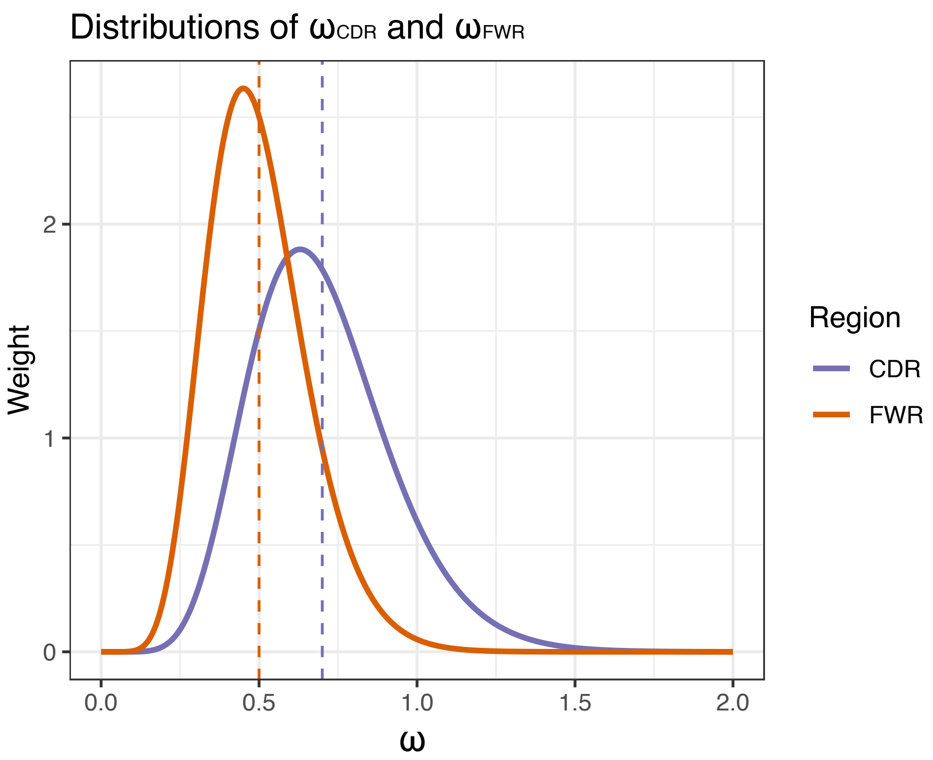

Figure S4a: Gamma distributions used to sample ω_{CDR} (purple) and ω_{FWR} (orange) values for each lineage for the simulations performed for **Table S4b**. The dotted lines indicate the mean of each distribution.

Parameter	Estimate	Bias	Variance	MSE
ω_{CDR}	Repertoire-wide	**0.0059**	**0.00072**	**0.00072**
	Mean individual, ≥ 2 seqs	210	440	22000
	Mean individual, ≥ 10 seqs	17	340	480
	Mean individual, ≥ 30 seqs	0.052	0.018	0.019
ω_{FWR}	Repertoire-wide	**-0.0096**	**0.000095**	**0.00012**
	Mean individual, ≥ 2 seqs	20	27	120
	Mean individual, ≥ 10 seqs	0.039	0.00047	0.00083
	Mean individual, ≥ 30 seqs	-0.012	0.0018	0.0018
κ	Repertoire-wide	**-0.0069**	**0.0013**	**0.0015**
	Mean individual, ≥ 2 seqs	4.1	20	87
	Mean individual, ≥ 10 seqs	0.02	0.005	0.0064
	Mean individual, ≥ 30 seqs	-0.0084	0.023	0.022
$h^{WR\underline{C}}$	Repertoire-wide	-0.062	**0.025**	**0.086**
	Mean individual, ≥ 2 seqs	2.8	29	160
	Mean individual, ≥ 10 seqs	**0.019**	0.1	0.1
	Mean individual, ≥ 30 seqs	-0.088	0.48	0.6
$h^{\underline{G}YW}$	Repertoire-wide	-0.15	**0.031**	0.84
	Mean individual, ≥ 2 seqs	2.4	43	250
	Mean individual, ≥ 10 seqs	**-0.081**	0.16	**0.39**
	Mean individual, ≥ 30 seqs	-0.17	0.55	1.6
$h^{W\underline{A}}$	Repertoire-wide	-0.033	**0.012**	**0.021**
	Mean individual, ≥ 2 seqs	4	30	170
	Mean individual, ≥ 10 seqs	0.061	0.07	0.1
	Mean individual, ≥ 30 seqs	**-0.021**	0.19	0.19
$h^{\underline{T}W}$	Repertoire-wide	-0.071	**0.005**	**0.01**
	Mean individual, ≥ 2 seqs	4.7	8.7	30
	Mean individual, ≥ 10 seqs	**0.048**	0.04	0.041
	Mean individual, ≥ 30 seqs	-0.092	0.089	0.095
$h^{SY\underline{C}}$	Repertoire-wide	**-0.017**	**0.00071**	**0.00081**
	Mean individual, ≥ 2 seqs	-2.7	2.9	5.5
	Mean individual, ≥ 10 seqs	-0.1	0.0038	0.0074
	Mean individual, ≥ 30 seqs	-0.1	0.024	0.027
$h^{\underline{G}RS}$	Repertoire-wide	0.016	**0.0019**	**0.002**
	Mean individual, ≥ 2 seqs	-6	7.9	21
	Mean individual, ≥ 10 seqs	-0.059	0.01	0.011
	Mean individual, ≥ 30 seqs	**-0.0046**	0.021	0.021
Mean	Repertoire-wide	**-0.04**	**0.01**	**0.11**
	Mean individual, ≥ 2 seqs	27	68	2500
	Mean individual, ≥ 10 seqs	1.9	38	53
	Mean individual, ≥ 30 seqs	-0.05	0.16	0.29

Table S4b: Performance of repertoire-wide and individual estimates in simulations with identical substitution parameters across lineages. Bias, variance, and mean squared error (MSE) of each substitution model parameter, or the mean across all parameters, were estimated using repertoire-wide estimates or the means of individual lineages containing at least 2, 10 and 30 sequences as indicated. The lowest (i.e. best) value of each comparison is shown in **bold**. Numbers are rounded to two significant digits.

True value(s)	Parameter	Estimate	Bias	Variance	MSE
Mean parameter value	ω_{CDR}	Repertoire-wide	**-0.024**	**0.0015**	**0.0017**
		Mean individual, ≥ 2 seqs	200	380	21000
		Mean individual, ≥ 10 seqs	8.9	150	180
		Mean individual, ≥ 30 seqs	0.035	0.046	0.045
	ω_{FWR}	Repertoire-wide	-0.05	**0.00021**	**0.00083**
		Mean individual, ≥ 2 seqs	20	17	110
		Mean individual, ≥ 10 seqs	0.033	0.00082	0.0011
		Mean individual, ≥ 30 seqs	**-0.000011**	0.0066	0.0064
Individual parameter values	ω_{CDR}	Repertoire-wide	**0.086**	**0.0015**	**0.05**
		Individual, ≥ 2 seqs	220	110000	140000
		Repertoire-wide	**0.093**	**0.0015**	**0.051**
		Individual, ≥ 10 seqs	8.3	6000	6000
		Repertoire-wide	0.14	**0.0015**	**0.047**
		Individual, ≥ 30 seqs	**0.082**	0.11	0.062
	ω_{FWR}	Repertoire-wide	**0.06**	**0.00021**	**0.026**
		Individual, ≥ 2 seqs	21	8000	9800
		Repertoire-wide	0.066	**0.00021**	0.024
		Individual, ≥ 10 seqs	**0.044**	0.048	**0.022**
		Repertoire-wide	0.047	**0.00021**	0.026
		Individual, ≥ 30 seqs	**-0.0062**	0.028	**0.0062**

Table S4c: Performance of repertoire-wide and individual estimates in simulations where ω varies across lineages. In the first eight rows, the mean of the true parameter distribution (**Figure S4a**; dotted lines) was compared to the repertoire-wide estimate and mean individual estimate obtained from lineages with sequence counts at or above 2, 10 and 30 sequences. The last twelve rows show how repertoire-wide and individual estimates compared to the true parameter values of individual lineages (**Figure S4a**; distributions). Namely, for all lineages containing at least the specified number of sequences, that lineage's true parameter value was compared to the repertoire-wide estimate (using all lineages regardless of size), and the estimate obtained from that lineage individually. The mean of bias, variance, and mean squared error (MSE) across all comparisons in all repetitions is reported in the last three columns. The lowest (i.e. best) value for each comparison shown in **bold**. Numbers are rounded to two significant digits.

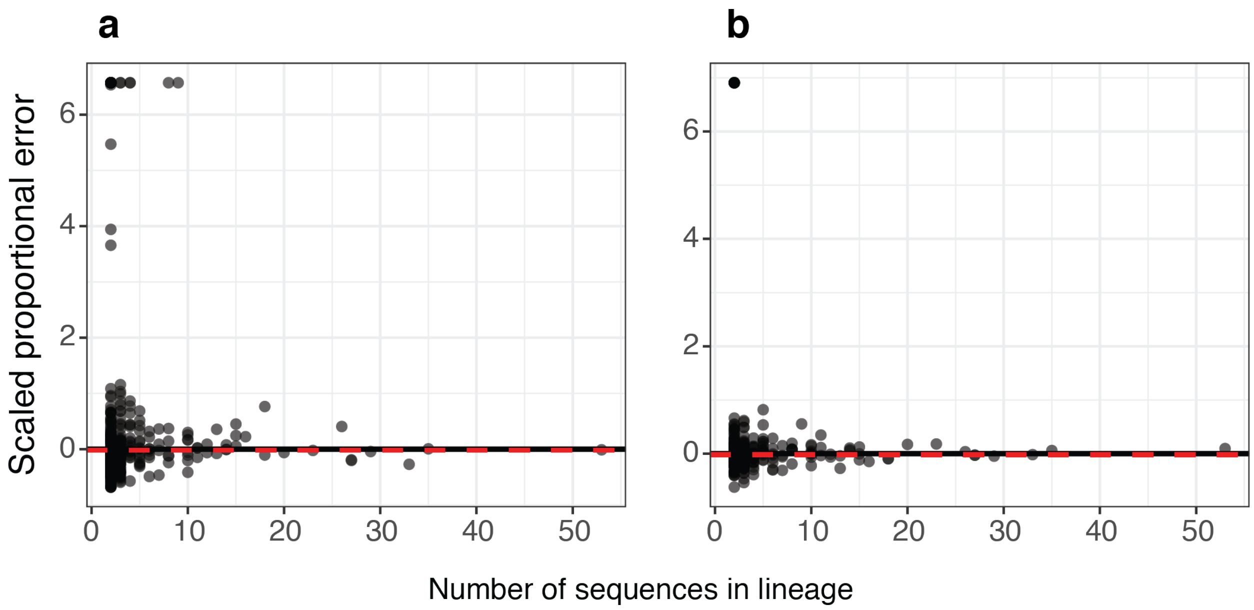

Figure S4d: Scaled proportional error of repertoire-wide and individual lineage estimates across their full range. Proportional error was scaled using the formula ln(*proportional error* + 2) – ln(2) to account for the fact that proportional error of ω could range from -1 to ∞. (**a**) Scaled proportional error in estimates of the ω_{CDR} parameter under the HLP19 model. (**b**) Scaled proportional error in estimates of the ω_{FWR} parameter under the HLP19 model. In both panels, the black dots show the values estimated from each individual lineage B cell lineage and the red lines shows the estimate obtained from all lineages combined using a repertoire-wide model. Data were generated from a simulated repertoire using tree topologies from subject 97 in the Age dataset and identical parameters among lineages. Note that this figure is the same data as in **Figure 1**, but plotted across its full range.

Section S5: Comparison of model fit on empirical datasets

The Akaike information criterion (AIC) is a means of scoring the quality of model fit to data that compares the number of freely estimated parameters to the maximum log likelihood calculated using the model (20). Lower AIC values indicate a better model fit. Here, we calculate AIC for GY94 (5, 6), HLP17 (1), and HLP19 (**Section S1**) models when our parameter estimation procedure (**Methods**: *Phylogenetic model parameter and topology estimation*) was applied to each subject of the Age dataset (**Table S5**). Because GY94 and HLP17 are expected to overestimate ω_{CDR} (**Section S3**), we also report ML estimates of ω_{CDR} for each model. In all subjects, both AIC and ω_{CDR} followed the pattern GY94 > HLP17 > HLP19, indicating HLP19 had both a better fit to the datasets and lower estimates of ω_{CDR}.

The GY94 model used 3 free parameters (κ, ω_{FWR}, and ω_{CDR}), HLP17 used 18 (κ, ω_{FWR}, ω_{CDR}, $h^{WR\underline{C}}$, $h^{\underline{G}YW}$, $h^{W\underline{A}}$, $h^{\underline{T}W}$, $h^{SY\underline{C}}$, $h^{\underline{G}RS}$, and three free base frequencies for each of three codon sites under the CF3X4 model (19), and HLP19 used 9 (κ, ω_{FWR}, ω_{CDR}, $h^{WR\underline{C}}$, $h^{\underline{G}YW}$, $h^{W\underline{A}}$, $h^{\underline{T}W}$, $h^{SY\underline{C}}$, $h^{\underline{G}RS}$). To make AIC values comparable among the three models, we altered the HLP17 and HLP19 models slightly by multiplying the partial likelihood of each possible codon at the root by the frequency of that codon ($\boldsymbol{\pi}$), as is typically done for reversible models (21). For GY94 these frequencies were the empirical estimates of codon frequencies under a CF3X4 model for each lineage within the repertoire, for HLP17 these frequencies were estimated by ML using the CF3X4 model and shared across all lineages within the repertoire, while for HLP19 these frequencies were estimated from the root sequence of each lineage ($\boldsymbol{\pi}^{\mathbf{g}}$ for FWR or CDR, **Section S1**).

	AIC			ω_{CDR}		
Subject	GY94	HLP17+$\boldsymbol{\pi}$	HLP19+$\boldsymbol{\pi}$	GY94	HLP17+$\boldsymbol{\pi}$	HLP19+$\boldsymbol{\pi}$
1	363603	360109	309005	0.99	0.77	0.69
3	607461	602820	513667	1.04	0.80	0.70
4	243193	241238	206345	1.03	0.83	0.74
8	733364	727169	619748	0.99	0.77	0.67
11	573867	567545	490976	0.86	0.67	0.60
13	472848	469177	398199	1.14	0.88	0.79
14	171315	170651	142884	1.00	0.77	0.67
15	247645	245197	208959	0.79	0.60	0.55
16	363741	360629	307737	1.07	0.86	0.75
18	453782	448849	387236	0.91	0.68	0.60
21	264276	261115	226276	0.79	0.59	0.52
23	108222	107762	90691	1.18	0.93	0.87
25	354698	351053	302490	1.00	0.78	0.69
26	461696	457367	392300	1.04	0.78	0.69
33	494143	488992	419978	1.02	0.78	0.69
34	434464	430241	369880	0.93	0.72	0.64
49	402052	398526	340069	0.98	0.77	0.69
54	532664	526649	455190	1.01	0.79	0.67
63	202962	201174	171439	0.95	0.72	0.63
64	281633	279516	237513	0.99	0.78	0.69
65	337095	333429	288345	0.99	0.76	0.69
73	217224	215238	186094	1.06	0.85	0.70
74	274756	272262	233127	1.06	0.79	0.67
83	312956	309557	266105	0.99	0.75	0.66
93	249065	247057	210139	1.09	0.83	0.72
95	216803	215498	181918	1.22	0.95	0.82
97	313170	308940	271512	0.77	0.61	0.53

Table S5: AIC values (rounded to the nearest integer) and ω_{CDR} estimates of all subjects in the Age dataset using the GY94, HLP17 and HLP19 substitution models, modified as detailed in **Section S5**.

Section S6: Simulation analyses based on empirical datasets

The goal of these simulation analyses is to test whether differences in the underlying tree topology and branch lengths of samples within the Age and Vaccine dataset could be responsible for observed trends between substitution model parameters and age in the Age dataset and time in the Vaccine dataset. As described in **Methods**: *Phylogenetic model parameter and topology estimation*, we first estimated maximum likelihood tree topologies, branch lengths, and shared values of ω, and κ for all lineages within each repertoire under the GY94 model. Codon base frequencies were set to their empirical estimates across all lineages within the repertoire using a CF3X4 model (19). For each lineage tree within each repertoire, we used the predicted germline sequence (see *Repertoire dataset processing* in **Section S1**) of the empirical data as the progenitor of each lineage. Masked codons (specified by 'NNN') were replaced with codons drawn from a random uniform distribution. These were primarily in the CDR3 region which was removed after simulations. Simulations were then performed using the procedure outlined in **Section S2**, using substitution parameters $\kappa = 2$, $\omega_{FWR} = 0.5$, $\omega_{CDR} = 0.7$, $h^{WR\underline{C}} = 4$, $h^{\underline{G}YW} = 6$, $h^{W\underline{A}} = 3$, $h^{\underline{T}W} = 1$, $h^{SY\underline{C}} = -0.6$, and $h^{\underline{G}RS} = -0.6$. These were chosen to be similar to those estimated from the Age dataset (**Figure 2**). We simulated 20 repertoire datasets for each sample in both datasets using this procedure and removed the CDR3 regions of all simulated sequences. We then repeated the parameter estimation procedure in **Methods**: *Phylogenetic model parameter and topology estimation* by first estimating tree topologies, branch lengths, and shared values of ω, and κ for all lineages within each repertoire under the GY94 model, and then estimating substitution parameters across all lineages using a repertoire-wide HLP19 model. For GY94

parameter estimation, codon base frequencies were set to their empirical estimates across all lineages within the repertoire using a CF3X4 model (19).

We first investigated whether our simulations could reproduce the observed negative relationship between age, sex, and estimates of $h^{W\underline{A}}$. For each repetition of our Age dataset of 27 subjects, we fitted a multiple linear regression model with age and sex as interaction variables against substitution rate biases of W$\underline{\text{A}}$ motifs (i.e. $h^{W\underline{A}}$). We compared the slope coefficients estimated from each of our simulated datasets to those observed in our empirical data. For all 20 repetitions the $h^{W\underline{A}}$ slope coefficents for males and females were closer to zero than their respective empirical estimates (**Figure S6a**).

We next investigated whether our simulations could reproduce the behavior of ω_{CDR} over the time course of influenza vaccination in our Vaccine dataset. As shown in **Figure S6c**, estimates of ω_{CDR} varied fairly constantly around a mean slightly above the true value (0.7) for the entire time course for all three subjects. For subjects 420IV and hu420139, we calculated the fold change in ω_{CDR} at day +7 compared to the pre-vaccine sample (-1 hour) for each simulation, and compared it to the empirical estimate. No simulation reproduced the observed fold change at day +7 in either subject (**Figure S6b**). As expected given its low sequence count, PGP1 day +14 had wide variation in ω_{CDR} estimate between repetitions. **Figure S6c** confirms that the simulation procedure reliably reproduced differences in mean tree length among repertoire datasets with little variation among repetitions.

We then tested whether our results in either the Age or Vaccine dataset could reproduce our observed negative relationship between ω and mean tree length. For each repetition of our Age and Vaccine datasets, we fit a log-linear regression model in which either ω_{CDR} or ω_{FWR} are modelled against the natural logarithm of mean tree length. We compared the slope coefficients estimated from each of our simulated datasets to those observed in our empirical data. For all 20 repetitions, slopes of both ω parameters from both the Age and Vaccine dataset were far lower than any slope parameters observed in the empirical Age or Vaccine datasets (**Figure S6d**). These results are further shown in **Figure S6e**, which shows these simulation results plotted as regressions and scatterplots.

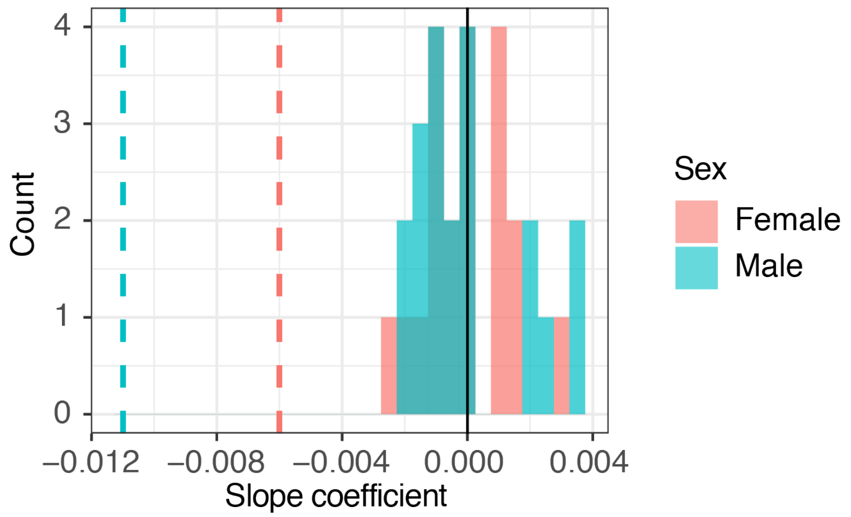

Figure S6a: Slope coefficients between $h^{W\underline{A}}$ and age for 20 null simulation repetitions of the Age dataset. Coefficients are colored by sex. Vertical dashed lines show the values observed in empirical data for their respective parameter and sex.

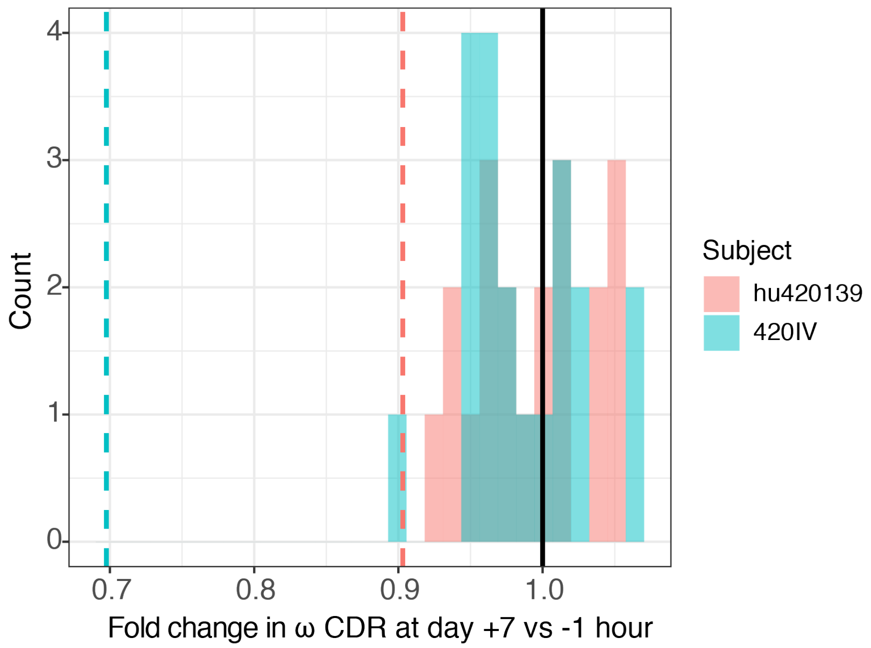

Figure S6b: Fold change in ω_{CDR} between day +7 and -1 hour (pre-vaccination) estimated for 20 null simulation repetitions for subjects hu420139 and 420IV (histograms) and empirical data (vertical lines).

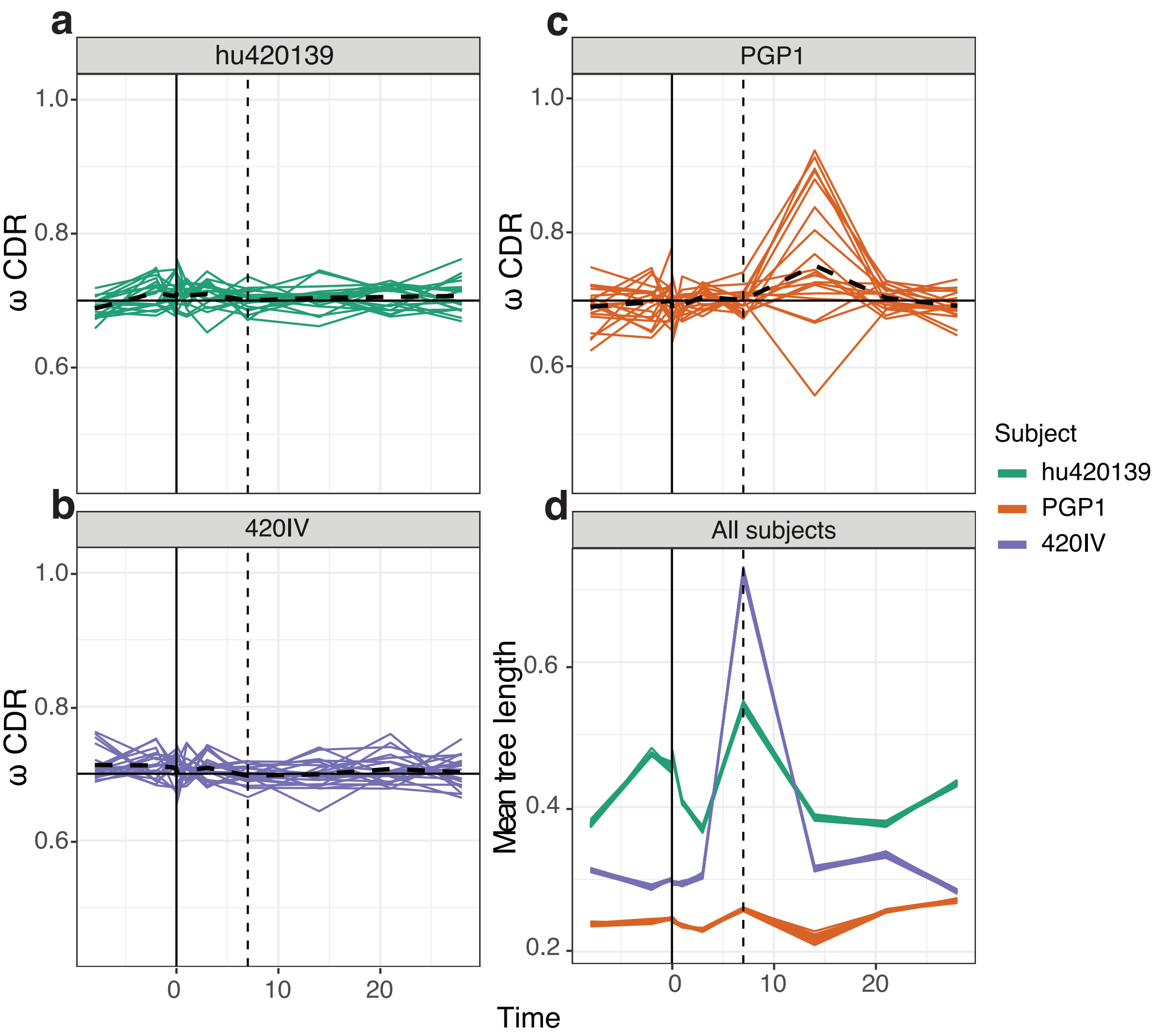

Figure S6c: **a-c**: plots of ω_{CDR} over time for each subject in the Vaccine dataset. Each line represents a repetition of the simulation procedure for a subject. The dashed black line shows the mean value across repetitions at a time point. The true value of ω_{CDR} in these simulations was 0.7, shown by the solid horizontal black line in each plot. **d**: plots of mean tree length over time for all subjects in the Vaccine dataset. Each line represents a repetition of the simulation procedure for a subject. These lines are closely overlaid due to the lack of variance in this parameter among repetitions.

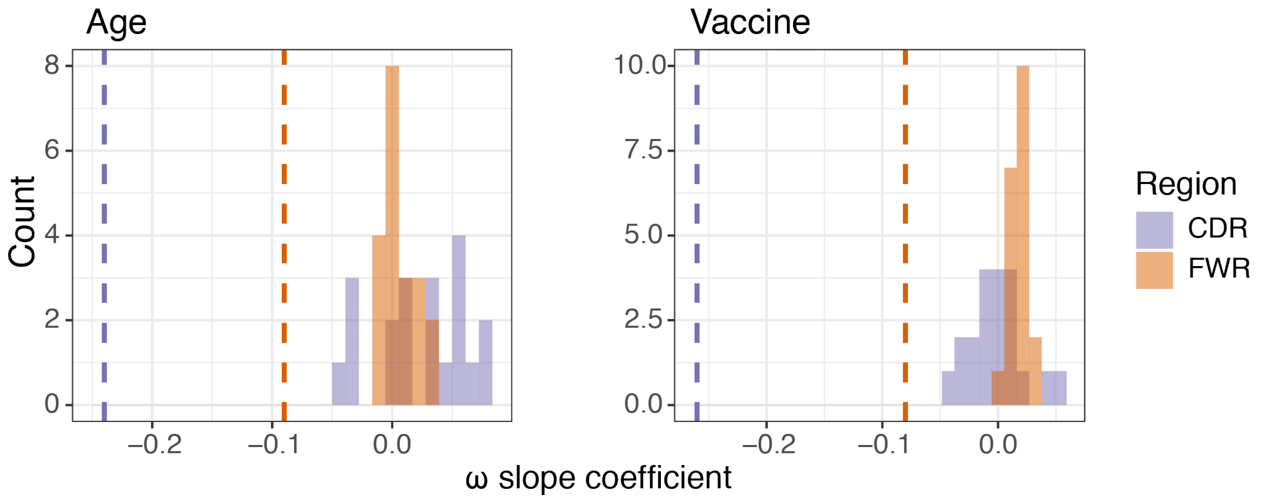

Figure S6d: Slope coefficients of log-linear regressions between ω and mean tree length in both the Age and Vaccine datasets among 20 simulation repetitions. Coefficients are colored by region (i.e. whether they represent ω_{CDR} or ω_{FWR}). Vertical dashed lines show the values observed in their respective empirical datasets.

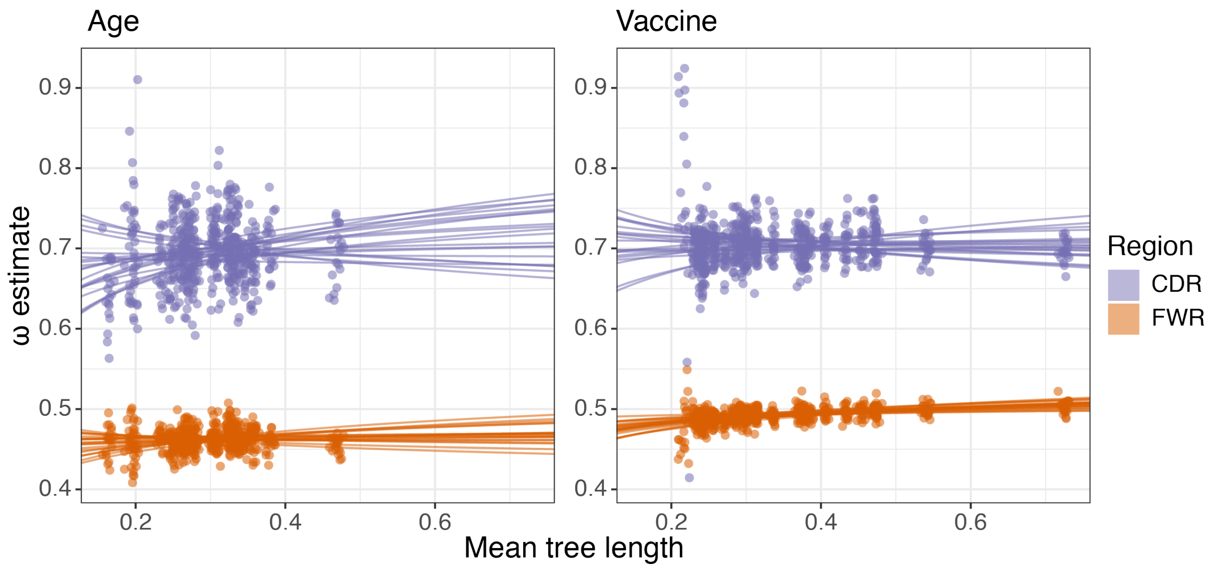

Figure S6e: Log-linear regressions between ω and mean tree length among 20 simulation repetitions of the Age and Vaccine datasets. These represent the same data as in **Figure S6c**. Coefficients are colored by region (i.e. whether they represent ω_{CDR} or ω_{FWR}). The true value for all simulations $\omega_{CDR} = 0.7$ and $\omega_{FWR} = 0.5$. The y axis is scaled identically between the two plots.

Section S7: Additional simulations using an empirical SHM model

As an additional validation, we also performed simulations based on the Age dataset using the S5F model within SHazaM (15). Simulations were performed under a modified version of Alakazam v0.2.11 (13) and SHazaM v1.9.0 (15) which are noted below and are available at: https://bitbucket.org/kleinstein/projects. These simulations for each clone were performed as follows:

1. For a given set of clonal sequences of at least two unique sequences, maximum parsimony lineage tree topology and branch lengths were estimated using *dnapars* from PHYLIP v3.697 (22), implemented in Alakazam v0.2.11(13). In contrast to the default behavior in Alakazam of estimating branch lengths using the Hamming distance between reconstructed intermediate sequences, branch lengths were estimated from PHYLIP as the estimated substitutions per site. Trees are also kept strictly binary and do not collapse zero-length branches in internal nodes.
2. Alakazam uses 'N' nucleotides to place all sequences within a clone into alignment with respect to IMGT numbering, which adds uninformative sites to the alignment. This means branch lengths taken directly from *dnapars* output – in units of substitutions per site – will be artificially low (this is not a problem in the default setting since *dnapars* branch lengths are not used directly). To correct for this, we re-scale branch lengths; namely, we multiply each branch length by the number of sites in the full alignment, and divide by the number of informative sites (codon sites with any non-N nucleotides in any data sequence) in the alignment.

3. Branch lengths are converted into the number of substitutions between nodes by multiplying each branch length by the number of informative sites in the germline sequence, and randomly rounding up or down based on difference from the nearest integer.
4. In contrast to the default behavior of SHazaM v1.9.0 (15), simulations begin with the clone's predicted germline sequence (GERMLINE_IMGT_D_MASK) from CreateGermlines.py from Change-O v0.4.3 (13). Mutations are added to the starting sequence based on their relative probability under the S5F model (15). Starting from the germline sequence at the root node of the tree, this process is repeated for each descendent node in the tree, with the number of mutations added between ancestor and child nodes equal to the branch length between them.
5. This process is repeated for all clones within a subject in the Age dataset.
6. These simulated datasets are converted into an IgPhyML-readable format using BuildTrees.py in Change-O v0.4.5 (13). We then repeat our parameter estimation procedure (see **Methods**:*Phylogenetic model parameter and topology estimation*) by first estimating maximum likelihood tree topologies, branch lengths, and repertoire-wide substitution parameters using the GY94 model, and then using these tree topologies to estimate maximum likelihood branch lengths and substitution model parameters under the HLP19 model.

For each simulation repetition of all 27 subjects, we fit a log-linear regression model in which either ω_{CDR} or ω_{FWR} are modelled against ln(mean tree length). We compared the slope coefficients estimated from each of our simulated datasets to those observed in our empirical

data. Of the 50 simulation repetitions performed, none showed a slope as negative as that observed between either ω_{CDR} or ω_{FWR} and mean tree length (**Figure S7a**). These results are further shown in **Figure S7b**, which shows these simulation results plotted as regressions and scatterplots. From this we conclude that even under this different simulation framework, which uses a model of SHM not fully captured by the HLP19 model, that the observed trend is not the result of model bias between tree length and ω, or selection of germline sequence.

We also compared performance of HLP17 and GY94 models on each of these simulated datasets. We repeated the parameter estimation procedure in **Methods**: *Phylogenetic model parameter and topology estimation* by first estimating tree topologies, branch lengths, and shared values of ω, and κ for all lineages within each repertoire under the GY94 model. For this initial GY94 parameter estimation, codon base frequencies were set to their empirical estimates across all lineages within the repertoire using a CF3X4 model (19). We then estimated substitution parameters across all lineages under repertoire-wide HLP19, HLP17, and GY94 models with separate ω parameters for FWR and CDRs (ω_{CDR} and ω_{FWR}, respectively). Our results largely reproduce those of **Section S3**. As expected, both HLP19 ω estimates are roughly centered at 1 (mean ω_{CDR} = 1.01 , mean ω_{FWR} = 1.01), while HLP17 tended to overestimate ω_{CDR} (mean ω_{CDR} = 1.11, mean ω_{FWR} = 1.0). GY94 tended to overestimate ω_{CDR} even more than HLP17, and underestimate ω_{FWR} (mean ω_{CDR} = 1.29, mean ω_{FWR} = 0.88). Overall, these confirm that HLP19 estimates of ω are not severely affected by alternative models of SHM context sensitivity, while estimates using HLP17 and GY94 models are more biased.

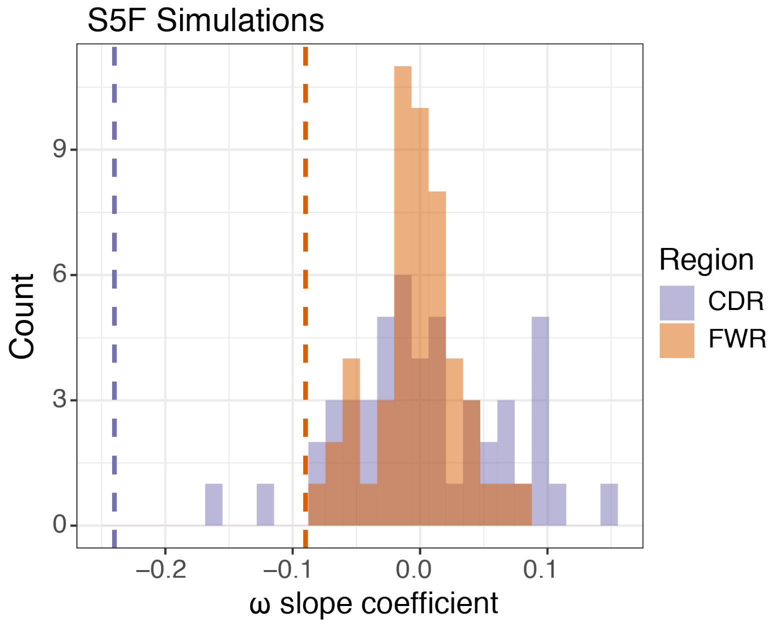

Figure S7a: Slope coefficients of log-linear regressions between HLP19 estimated ω (ω_{CDR} =purple, ω_{FWR} =orange) and mean tree length among 50 simulation repetitions of the Age dataset. Vertical dashed lines show the values observed in their respective empirical datasets.

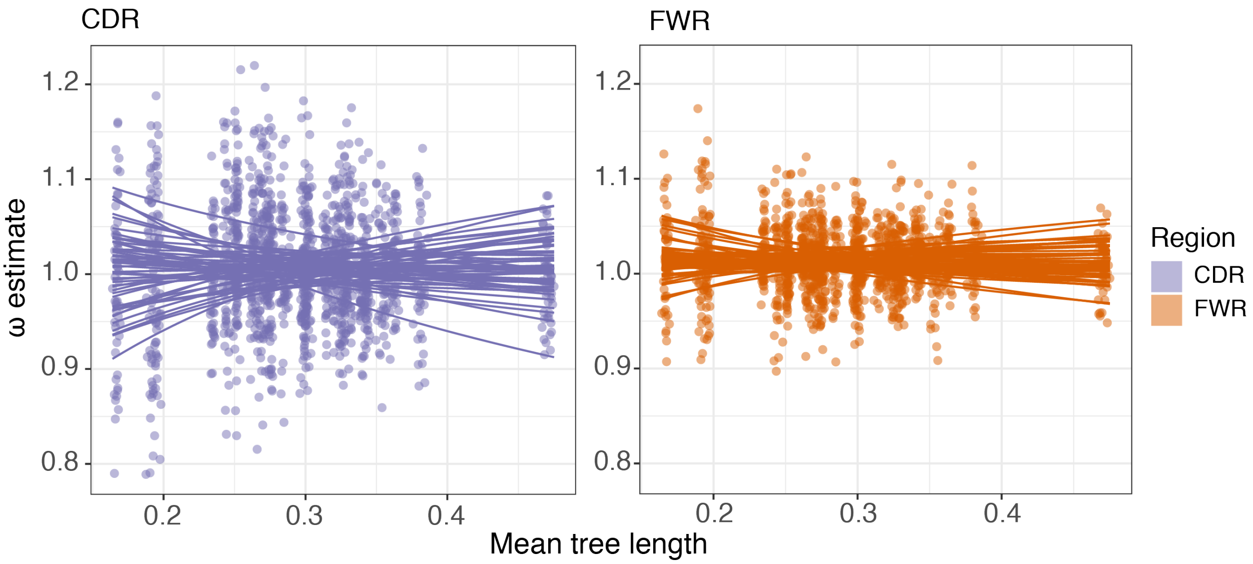

Figure S7b: Log-linear regressions between HLP19 estimated ω (ω_{CDR} =left panel, ω_{FWR} =right panel) and mean tree length among 50 simulation repetitions of the Age dataset. These represent the same data as in **Figure S7a**. The Y axis is scaled identically in the two plots.

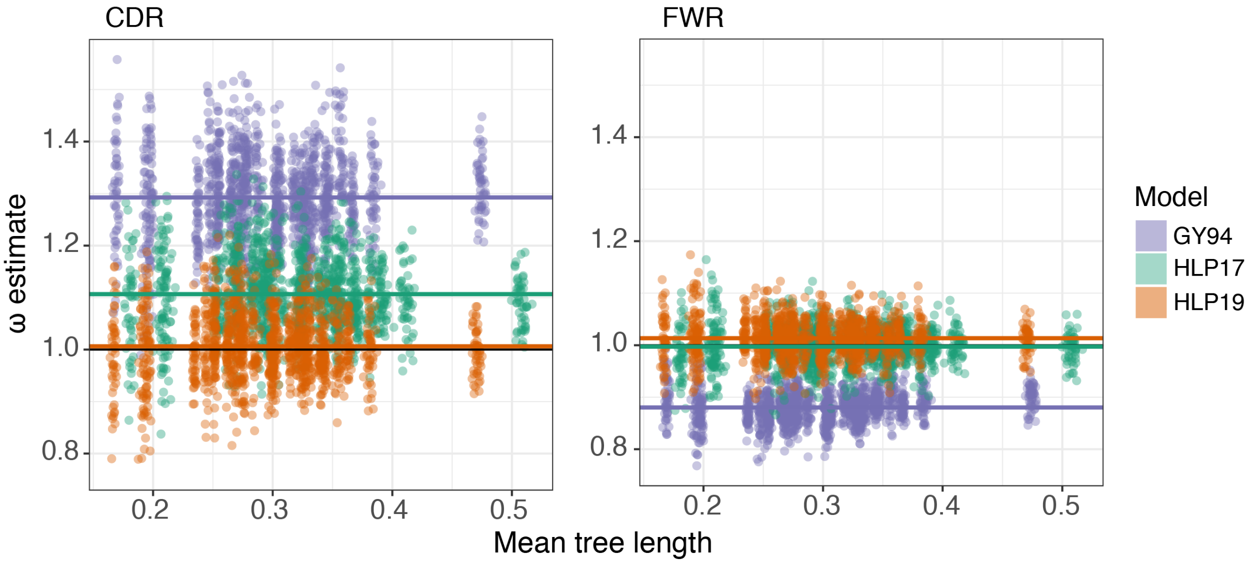

Figure S7c: Estimates of ω_{CDR} (left panel) and ω_{FWR} (right panel) among 50 simulation repetitions of the Age dataset. These represent the same data as in **Figure S7a** and **S7b**. The parameters ω_{CDR} and ω_{FWR} were estimated for each simulated dataset using the GY94 (purple), HLP17 (green), and HLP19 (orange) models. Mean estimates for each model are shown as solid lines. A separate solid black line shows the expected value ω=1.0.

Section S8: Streams plots of clone sizes over time

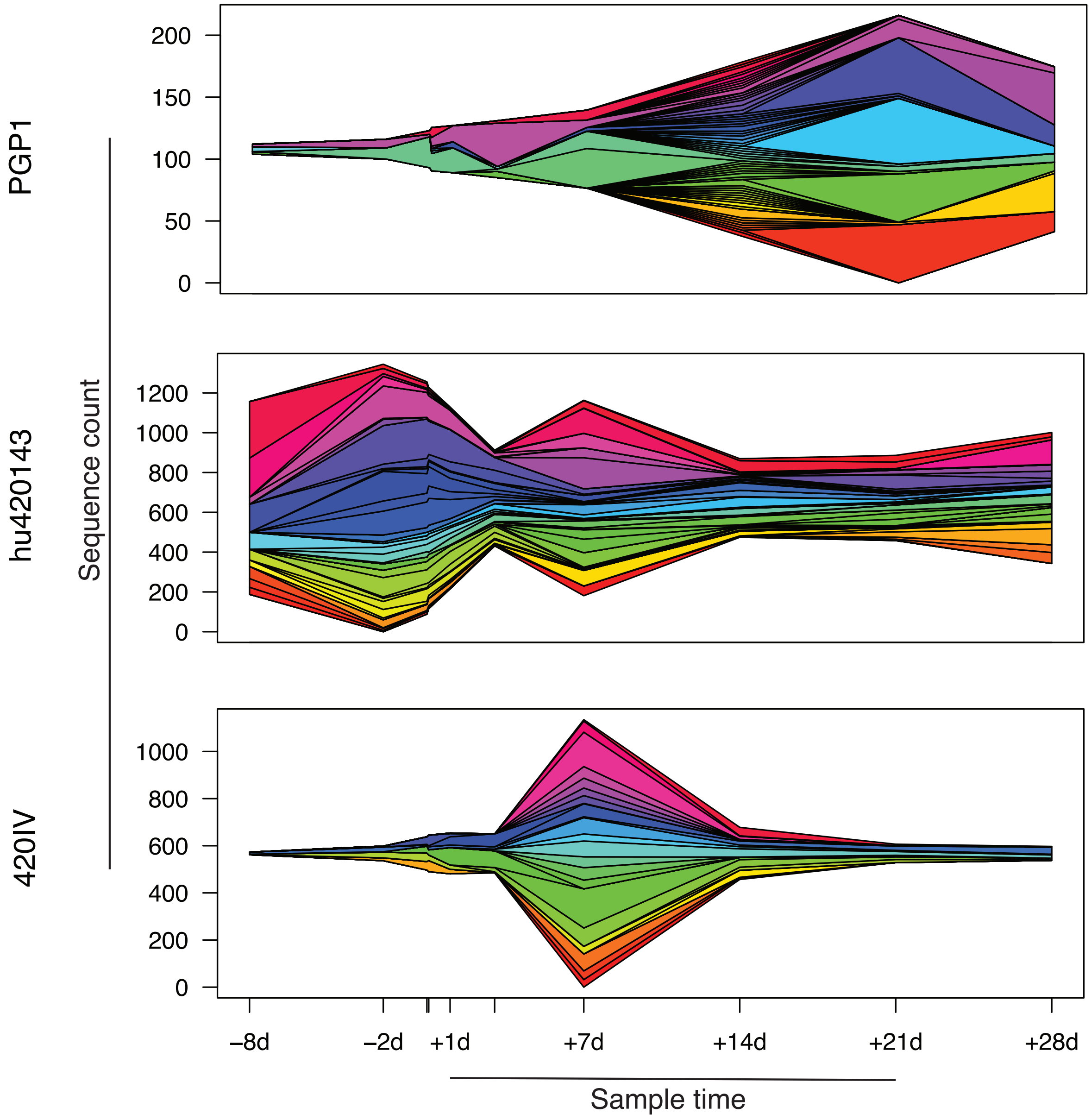

Figure S8: Stream plots showing sequence count of large (≥1% of total repertoire sequences) clonal lineages at multiple time-points pre- and post-influenza vaccination of three individuals (PGP1, hu420143 and 420IV). Counts are defined by the number of unique sequences determined using the full V(D)J sequence of the BCR.

Section S9: Estimates of ω_{CDR} for lineages of different sizes

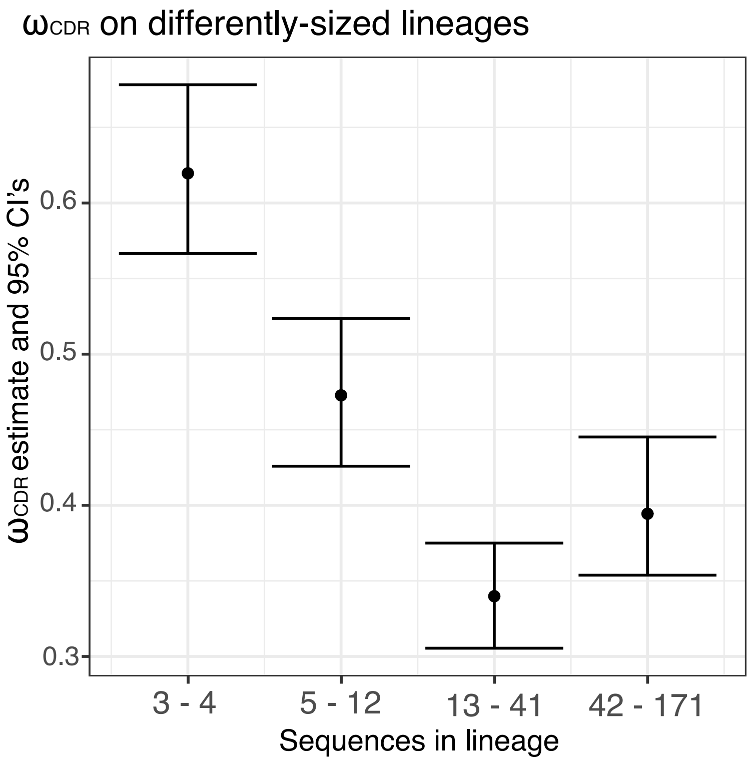

Figure S9: Estimates of ω_{CDR} for lineages of different sizes from the same repertoire (subject 420IV, day +7 post-vaccination). Samples on the x-axis show approximate quartiles of sequence count in the repertoire, with larger lineages on the right and smaller lineages on the left. Lineages within their respective size constraints were grouped together and ω_{CDR} was estimated for each group using the HLP19 model and the same parameter estimation procedure described in **Methods**: *Phylogenetic model parameter and topology estimation*. These results show that lineages with more sequences tended to have lower ω_{CDR} estimates compared to lineages with fewer sequences, though this trend is not monotonic. Sequence counts represent number of unique sequences in a lineage before the CDR3 region is masked.

Section S10: 95% confidence intervals of estimates of ω_{CDR} and ω_{FWR} for subjects in the Age dataset

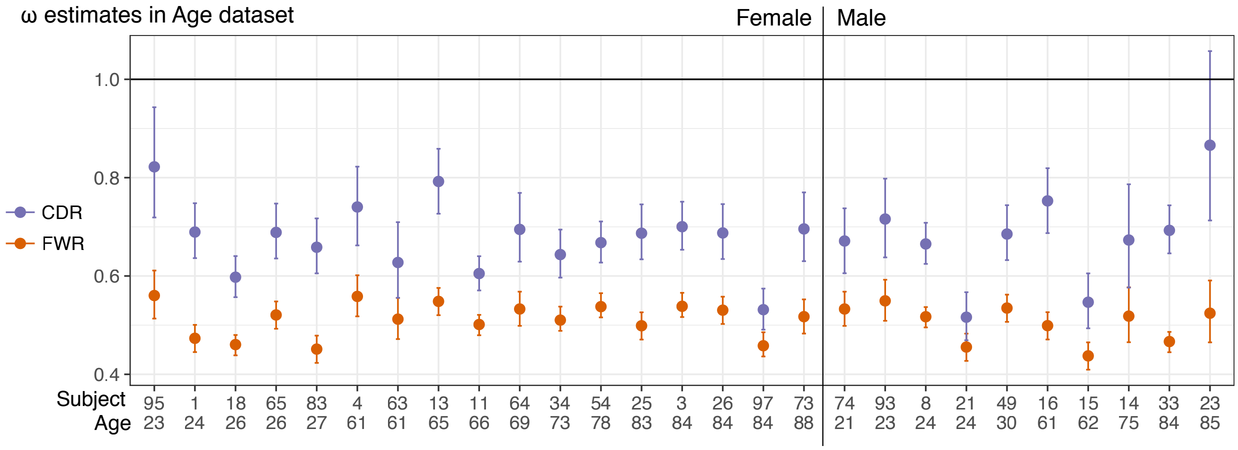

Figure S10: Maximum likelihood estimates (points) and 95% confidence intervals of ω_{CDR} (purple) and ω_{FWR} (orange) for each subject in the Age dataset, ordered within sex by age (in years) at the time of sampling.

Supplemental References

1. Hoehn KB, Lunter G, Pybus OG (2017) A Phylogenetic Codon Substitution Model for Antibody Lineages. *Genetics* 206(1):417–427.

2. Peled JU, et al. (2008) The Biochemistry of Somatic Hypermutation. *Annu Rev Immunol* 26(1):481–511.

3. Lio P, Goldman N (1998) Models of molecular evolution and phylogeny. *Genome Res* 8(12):1233–1244.

4. Sheng Z, et al. (2016) Effects of Darwinian Selection and Mutability on Rate of Broadly Neutralizing Antibody Evolution during HIV-1 Infection. *PLOS Comput Biol* 12(5):e1004940.

5. Goldman N, Yang Z (1994) A codon-based model of nucleotide substitution for protein-coding DNA sequences. *Mol Biol Evol* 11(5):725–736.

6. Nielsen R, Yang Z (1998) Likelihood Models for Detecting Positively Selected Amino Acid Sites and Applications to the HIV-1 Envelope Gene. *Genetics* 148(3):929–936.

7. Heiden JAV, et al. (2017) Dysregulation of B Cell Repertoire Formation in Myasthenia Gravis Patients Revealed through Deep Sequencing. *J Immunol*:1601415.

8. Yang Z (1994) Estimating the pattern of nucleotide substitution. *J Mol Evol* 39(1):105–111.

9. Kaehler BD, Yap VB, Huttley GA (2017) Standard Codon Substitution Models Overestimate Purifying Selection for Nonstationary Data. *Genome Biol Evol* 9(1):134–149.

10. Wang C, et al. (2013) Effects of Aging, Cytomegalovirus Infection, and EBV Infection on Human B Cell Repertoires. *J Immunol*:1301384.

11. Ye J, Ma N, Madden TL, Ostell JM (2013) IgBLAST: an immunoglobulin variable domain sequence analysis tool. *Nucleic Acids Res* 41(W1):W34–W40.

12. Giudicelli V, Chaume D, Lefranc M-P (2005) IMGT/GENE-DB: a comprehensive database for human and mouse immunoglobulin and T cell receptor genes. *Nucleic Acids Res* 33(suppl 1):D256–D261.

13. Gupta NT, et al. (2015) Change-O: a toolkit for analyzing large-scale B cell immunoglobulin repertoire sequencing data. *Bioinformatics* 31(20):3356–3358.

14. Gupta NT, et al. (2017) Hierarchical Clustering Can Identify B Cell Clones with High Confidence in Ig Repertoire Sequencing Data. *J Immunol* 198(6):2489–2499.

15. Yaari G, et al. (2013) Models of Somatic Hypermutation Targeting and Substitution Based on Synonymous Mutations from High-Throughput Immunoglobulin Sequencing Data. *Front Immunol* 4. doi:10.3389/fimmu.2013.00358.

16. Laserson U, et al. (2014) High-resolution antibody dynamics of vaccine-induced immune responses. *Proc Natl Acad Sci* 111(13):4928–4933.

17. Vander Heiden JA, et al. (2014) pRESTO: a toolkit for processing high-throughput sequencing raw reads of lymphocyte receptor repertoires. *Bioinformatics* 30(13):1930–1932.

18. Lefranc M-P, Lefranc G (2001) *The Immunoglobulin FactsBook* (Academic Press).

19. Kosakovsky Pond S, Delport W, Muse SV, Scheffler K (2010) Correcting the Bias of Empirical Frequency Parameter Estimators in Codon Models. *PLoS ONE* 5(7):e11230.

20. Akaike H (1974) A new look at the statistical model identification. *IEEE Trans Autom Control* 19(6):716–723.

21. Felsenstein J (1981) Evolutionary trees from DNA sequences: A maximum likelihood approach. *J Mol Evol* 17(6):368–376.

22. Felsenstein J (2002) *{PHYLIP} (Phylogeny Inference Package) version 3.6a3*.
